# Modulation of Solvent Properties Using Imidazolium-Based Ionic Liquids: Effects on the Thermodynamics of PEO–PPO–PEO Triblock Copolymer Aggregation

**DOI:** 10.1021/acsomega.5c07226

**Published:** 2025-09-29

**Authors:** Álvaro Javier Patiño-Agudelo, Guilherme Max Dias Ferreira, Gabriel Max Dias Ferreira, Yara Luiza Coelho, Isabela A. Marques, Jaqueline P. Rezende, Ana Clarissa dos Santos Pires, Luis Henrique Mendes da Silva

**Affiliations:** † Advanced Thermokinetics of Molecular Systems (ATOMS) Group, Chemistry Department, 28120 Federal University of Viçosa, Viçosa, Av. P. H. Rolfs s/s, Viçosa, Minas Gerais 36570900, Brazil; ‡ Department of Physical Chemistry, Institute of Chemistry, University of Campinas, UNICAMP, Campinas, São Paulo 13083-970, Brazil; § Group of Material, Interface, and Solutions (MatIS), Department of Chemistry, 67739 Universidade Federal de Lavras, Campus Universitário, Lavras, Minas Gerais 37200000, Brazil; ∥ Department of Chemistry, Federal University of Ouro Preto, Campus Universitário, Ouro Preto, Minas Gerais 35400000, Brazil; ⊥ Chemistry Institute, Federal University of Alfenas, Campus Universitário, Alfenas, Minas Gerais 37130000, Brazil; # Department of Food Science, Federal University of Lavras, Campus Universitário, Lavras, Minas Gerais 37200000, Brazil; ¶ Applied Molecular Thermodynamics (THERMA), Department of Food Technology, Federal University of Viçosa, Av. P. H. Rolfs s/s, Viçosa, Minas Gerais 36570900, Brazil

## Abstract

Differential scanning calorimetry, isothermal titration calorimetry, fluorescence spectroscopy, and dynamic light scattering were used to investigate the temperature- and concentration-induced aggregation of PEO–PPO–PEO triblock copolymers (L31, L81, L64, P123, and F127) in aqueous solutions of 1-butyl-3-methylimidazolium halides (C_4_mimX, X = Cl^–^ or Br^–^) and sodium chloride (NaCl), establishing a comparative energetic and structural framework for understanding how they modulate triblock copolymer self-assembly. The critical micellization temperature decreased in the presence of cosolutes following the order NaCl < C_4_mimCl < C_4_mimBr, while the critical micellar concentration increased with C_4_mimX (C_4_mimCl < C_4_mimBr) and decreased with NaCl. Micellization was less endothermic with C_4_mimX but more endothermic with NaCl. These trends are attributed to the solvation of the PPO block by C_4_mimX (Cl^–^ > Br^–^), which displaces water molecules and results in a higher partial molar enthalpy. This effect is modulated by the EO fraction of the copolymer, as revealed by principal component analysis. Such interactions play distinct roles in the desolvation of triblock copolymer unimers during micelle formation and in the transition from spherical to rod-like micelles. The results provide a holistic energetic understanding of the modulation of energy by ionic liquids during triblock copolymer aggregation.

## Graphical abstract

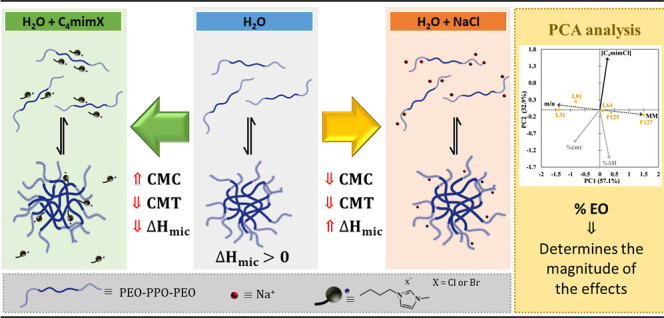

## 1 Introduction

Understanding the cooperative aggregation of amphiphilic compounds in aqueous solutions is key to the development of several technologies involving processes determined by weak interactions in the fields of medicine, chemistry, and biochemistry. Among the diverse cooperative aggregation processes, triblock copolymer micellization has shown great potential for application in various fields, such as gene^1^ and drug^2^ delivery, cell protection,^3^ nanoparticle synthesis,^4^ three-dimensional (3D) printing of irreversible hydrogel constructs,^5^ demulsifiers,^6^ and solubility of hydrophobic molecules in aqueous solutions.^7^

The most commonly used triblock copolymers are those formed by poly­(ethylene oxide) (PEO) and poly­(propylene oxide) (PPO), with the general chemical structure (EO)_*n*_–(PO)_*m*_–(EO)_*n*_, where *n* and *m* are the average number of ethylene oxide (EO) and propylene oxide (PO) units, respectively, in the macromolecule (unimer). Above a critical micellar concentration (CMC) or critical micellization temperature (CMT), triblock copolymer unimers aggregate, forming micelles at a fixed pressure.^8^ Micellization involves a delicate balance of energy associated with the formation and rupture of different intermolecular interactions, which can be strongly modulated by the properties of the solvent.^9–14^ A modern solvent with the capacity to control the thermodynamics and morphology of different cooperative processes is a mixture of water and imidazolium-based ionic liquids (ILs).^15^ Some IL structures in combination with water can modulate weak interactions that determine the aggregation processes, such as hydrophobic, electrostatic, van der Waals, and hydrogen bond (in addition to steric effects) interactions.^16–21^

Triblock copolymer micellization has been investigated in water-imidazolium IL mixtures since the work of Zheng et al.^22^ in 2007, where the effect of the concentration of 1-butyl-3-methylimidazolium bromide (C_4_mimBr) on the micellization of P104, (EO)_27_–(PO)_61_–(EO)_27_ (5% m/v), was evaluated using different techniques. An increase in the concentration of C_4_mimBr decreased the CMT and the size of the P104 micelles. These effects were associated with hydrophobic interactions between the C_4_mim^+^ cation and PPO blocks of the copolymer, with the incorporation of C_4_mimBr ionic pairs inside the micellar aggregates. In 2008 and 2009, Bhattacharyya et al.^23,24^ monitored the behavior of P123 micelles, (EO)_20_–(PO)_70_–(EO)_20_ (5 wt %), in solutions of 1-pentyl-3-methylimidazolium (C_5_mim^+^) with tetrafluoroborate (BF_4_^–^) or bromide (Br^–^), using dynamic light scattering (DLS) and spectroscopic analysis. They determined that at 0.3 mol L^–1^ IL, the C_5_mimBF_4_ (or C_5_mimBr) entered the hydrophobic PPO core of P123 micelles, and at 0.9 mol L^–1^, both ILs were embedded in the core and in the corona of the micelles. However, at high concentrations of the IL with BF_4_^–^, the aggregate hydrodynamic diameter (*D*_H_) decreased, and the opposite effect was observed for Br^–^; both effects were explained by ion hydration.

In 2012, Parmar et al.^25^ examined the cloud point (CP) and *D*_H_ of P103, (EO)_17_–(PO)_60_–(EO)_17_ (5%), in aqueous solutions of 1-alkyl-3-methylimidazolium tetrafluoroborates (C_*n*_mimBF_4_, *n* = 4, 6, or 8), through scattering and nuclear magnetic resonance techniques. Interestingly, while an increase in the concentration of C_4_mimBF_4_ or C_6_mimBF_4_ raised the CP and *D*_H_ values of P103 micelles, a higher content of C_8_mimBF_4_ produced the opposite effect. The effect promoted by the increase of the IL’s lateral chain length was explained by the compact alignment of the IL chains in the micelle core.

Between 2014 and 2017, Venkatesu, Reddy, and Umapathi^26–28^ evaluated the micellization of F108, (EO)_132_–(PO)_50_–(EO)_132_ (7 mg mL^–1^), induced by temperature increase in water–IL systems using multiple characterization methods. They also evaluated the effect of IL concentration (up to 15 mg mL^–1^) with different structures on micellization, combining: (i) the anions of the kosmotropic series (HSO_4_^–^ > CH_3_COO^–^ > Cl^–^ > I^–^ > BF_4_^–^ > SCN^–^) with the C_4_mim^+^ cation; (ii) the 1-allyl-3-methylimidazolium (Amim^+^) and 1-benzyl-3-methylimidazolium (Bzmim^+^) cations with Cl^–^; and (iii) 1-alkyl-3-methylimidazolium cations with different lateral chain lengths (C_*n*_mim, *n* = 2, 4, 6, or 10) with Cl^–^. The results showed that (i) the anion effect followed the well-known Hofmeister series and the CMT decreased with increasing IL, which was explained based on the charge and size of the anions and the weak ionic pair interactions forming the IL; (ii) the cation effect decreased the CMT with increasing IL concentration; this effect was more pronounced for Bzmim^+^ than Amim^+^, which was associated with the size and charge of the cations, beyond the presence of dipole–ion interactions between PPO blocks and IL ions; (iii) the increase in the lateral chain length of ILs decreased the CMT of the copolymer due to the insertion of ILs in the F108 micelles and the kosmotropicity of the IL cations.

Lunagariya et al.^29^ evaluated the effect of increasing the concentrations of 1-octyl-3-methylimidazolium halides (C_8_mimX, X = Cl^–^, Br^–^, or I^–^) and 1-alkyl-3-methylimidazolium chlorides (C_*n*_mimCl, *n* = 4, 6, or 8) on the micellization of F127, (EO)_100_–(PO)_70_–(EO)_100_, using DLS, surface tension, and viscosity at 303.15 K. The DLS results showed that in the presence of ILs (with different halides and alkyl chain lengths), the hydrodynamic radii (*R*_H_) of F127 micelles slightly decreased when compared to the value obtained in pure water. Moreover, the CMC values of F127 increased with the increasing kosmotropicity of the IL cation, which was attributed to the increase in the solubility of PPO (and PEO) in the solvent mixture.

He et al.^30^ and Zhang et al.^31^ evaluated the micellization of P123 in water–IL systems using small-angle neutron scattering, isothermal titration calorimetry (ITC), and pyrene fluorescence. They found that an increase in C_4_mimBF_4_ concentration (in the range of 0–1 mol L^–1^) increased the CMC and made the micellization process enthalpically less favorable, suggesting that the C_4_mim^+^ cations solvating the PEO and PPO blocks were involved in hydrophobic interactions and hydrogen bondings. Finally, Luo et al.,^32^ in 2022, studied the effect of C_*n*_mimBr with large lateral alkyl chain (*n* = 8, 10, 12, or 14) on the *D*_H_ of F127 aggregates using DLS, zeta potential, cryogenic transmission electron microscopy, and molecular dynamic simulations. C_*n*_mimBr acted as a cosurfactant performing simultaneously hydrophobic and hydrogen bond interactions with the PPO and PEO blocks, respectively, which promoted the decrease of *D*_H_. The compilation of all the information mentioned here concerning the effects of ILs on triblock copolymer aggregation is summarized in Table S1.

Although many studies have demonstrated that water–IL mixtures affect the triblock copolymer micellization process by evaluating parameters such as the CMC, CMT, CP, and *D*_H_, there have been no studies reporting the systematic comparison among thermodynamic parameters of aggregation induced by temperature and concentration changes of triblock copolymers with different structures in water–IL mixtures as well as comparing the effects of ILs and traditional electrolytes on micellization. A systematic study evaluating these aspects can help to systematize the main structural property of the triblock copolymers that determines the effect of the IL on the aggregation process.

In this study, we examined the effect of different concentrations of 1-butyl-3-methylimidazolium halides (C_4_mimCl or C_4_mimBr, Figure S1) and sodium chloride (NaCl) on the micellization of PEO–PPO–PEO triblock copolymers with different molar masses (MM) and hydrophilic/hydrophobic balances. Analyses were performed using differential scanning calorimetry (DSC), ITC, steady-state fluorescence spectroscopy, and DLS. Principal component analysis (PCA) was used to evaluate the main properties of the copolymer that determined the effect of the IL.

## 2 Materials and Methods

### 2.1 Materials

Table 1 contains the chemicals used in this work and their specifications. All of these compounds were used without further purification. Deionized water (18 MΩ cm at 298.2 K) from a Milli-QII reverse osmosis system (Millipore, USA) was used to prepare all solutions.

**Table 1:** Chemicals, Molar Masses (MM), Chemical Formulas, Purities, and Suppliers of the Chemicals Used in This Work^a^

chemicals	$\frac{MM}{g{\;mol}^{-1}}$	chemical formula	$\frac{purity}{\%}$	supplier
C_4_mimCl	174.67	C_8_H_15_ClN_2_	95.0	Sigma-Aldrich (USA)
C_4_mimBr	219.12	C_8_H_15_BrN_2_	97.0	Sigma-Aldrich (USA)
pyrene	202.25	C_16_H_10_	99.0	Sigma-Aldrich (USA)
NaCl	58.44	NaCl	99.0	ISOFAR (Brazil)
L31	1100	(EO)_1_–(PO)_18_–(EO)_1_		Sigma-Aldrich (USA)
L81	2800	(EO)_3_–(PO)_42_–(EO)_3_		Sigma-Aldrich (USA)
L64	2900	(EO)_13_–(PO)_30_–(EO)_13_		Sigma-Aldrich (USA)
P123	5800	(EO)_20_–(PO)_70_–(EO)_20_		Sigma-Aldrich (USA)
F127	12600	(EO)_106_–(PO)_70_–(EO)_106_		Sigma-Aldrich (USA)

a (i) The notation of triblock copolymer is formed by one letter and numbers. The letter is associated with the state of the pure triblock copolymer: liquid (L), paste (P), and flakes (F). The first one or two numbers are related to the MM of the PO block and the last number to the mass percentage of the EO block in the unimer. For instance, for F127, F corresponds to flakes, 12 is equivalent to ∼4000 g mol^–1^ of PO in the unimer, and 7 indicates that 70% of the MM of the F127 unimer are EO units (for more information, see the “Pluronic grid” in the work of Alexandridis and Hatton).^8^ (ii) The abbreviation used to the IL is C_*n*_mimX, i.e., 1-butyl-3-methylimidazolium chloride is C_4_mimCl, where the subscript “4” represents the number of carbons in the lateral chain of the aromatic ring, “mim” is the imidazolium cation, and X is the anion Cl^–^. (iii) The reported copolymer molar masses are averages, not exact values.

### 2.2 Sample Preparation and Density Measurements

All chemicals were weighed using an analytical balance with an uncertainty of ±0.001 g to prepare the stock solutions of triblock copolymer and IL (or NaCl), which were adequately mixed and diluted using precalibrated volumetric pipettes. Triblock copolymer concentrations were in % m/m, and IL (or NaCl) concentrations were in % mol/mol. To convert mass concentration to volume concentration, either to compare or determine the thermodynamic parameters, density measurements were made using a vibrating-tube densitometer (Anton Paar DMA 5000 M, Graz-Austria) with an uncertainty of ±0.000007 g/cm^3^, at 298.15 ± 0.01 K. The equipment calculated the density (ρ) from the quotient of the period of oscillation of the *U*-tube and the reference oscillator using eq 1

1 $$\rho =AQ^{2}f_{1}-Bf_{2}$$

where *A* and *B* are the apparatus constants, *f*_1_ and *f*_2_ are the correction terms for viscosity and temperature, and *Q* is the quotient of the period of oscillation of the *U*-tube divided by the period of oscillation of the reference oscillator.

### 2.3 Fluorescence Measurements

Fluorescence spectra of pyrene in triblock copolymer + electrolyte + water mixtures (or triblock copolymer + water) were obtained in a Cary Eclipse fluorescence spectrophotometer (Agilent Technologies, USA), equipped with an intense xenon flash lamp as a light source and with a thermostatic bath for temperature control. Experiments were performed at 283.2, 298.2, and 308.2 ± 0.1 K. The pyrene concentration in each solution was 1 μmol L^–1^ to prevent the formation of excimers in solution containing triblock copolymer in the unimeric state. The pyrene excitation wavelength was 336 nm, with both excitation and emission slit widths set to 5.0 nm, the photomultiplier tube voltage set to 650 V, and the spectra collected between 360 and 410 nm.

For each measurement, 3 mL of solvent was placed in the fluorometer cell and stirred at 100 rpm. Subsequently, injections with defined volumes of triblock copolymer solution, prepared by using calibrated micropipettes, were performed. After each injection, the mixture was allowed to equilibrate for 3 min before measuring the pyrene emission spectrum of the new mixture. The next titration was carried out at the fourth minute.

The polarity of the microenvironment around the pyrene was assessed by the intensity ratio between the first (*I*_1_ at 373 nm) and third (*I*_3_ at 384 nm) vibronic bands in the pyrene spectrum. Triplicate of spectra was obtained for each solution, and the emission intensity at each wavelength was the average of the intensities. The sigmoidal profile obtained was fitted using eq 2 ^33^

2 $$\frac{I_{1}}{I_{3}}=\frac{A_{1}-A_{2}}{1+e^{\frac{\left(x-x_{0}\right)}{dx}}}+A_{2}$$

where *A*_1_ and *A*_2_ are the upper (associated with the unimeric state) and lower (associated with the micellar state) limits of the sigmoidal fit, respectively, and *x*_0_ is the inflection point of the curve, defined as CMC, where the probability of additional unimers to remain in the bulk or enter in a micelle is 50%. The CMC value (in mol L^–1^) obtained was used to determine the standard Gibbs free energy change of micellization (Δ*G*_mic_^o^) using the following equation^34^

3 $${\Delta G}_{mic}^{o}=RT\ln CMC$$

where *R* and *T* are the gas constant and the absolute temperature, respectively.

Experiments evaluating the intrinsic fluorescence emission of the imidazolium ring in the C_4_mim^+^ cation (3.0% mol/mol), in the absence or presence of the P123 (0.1% m/m), were also conducted following the methodology proposed by Paul et al.^35^ The excitation wavelength was between 280 and 410 nm, and the spectra were collected up to 650 nm.

### 2.4 Calorimetric Measurements

#### 2.4.1 Differential Scanning Calorimetry

DSC measurements were performed in a Nano DSC equipped with two fixed platinum capillary cells (reference and sample) containing 300 μL of active volume each. The equipment was manufactured by TA Instruments (New Castle, USA) and controlled by DSC Run dedicated software. All solutions were degassed at a Nalgene degassing station (TA Instruments) before measurements. To obtain the DSC thermograms for analysis, two experiments were performed for each system. In the first one, both calorimetric cells were filled with the solvent (pure water or water + cosolute mixture) to obtain a baseline. In the second one, the solvent in the sample cell was replaced with a triblock copolymer solution prepared in the same solvent used to fill the reference cell. Both experiments were conducted with two scans, heating and cooling, in the following conditions: pressure of 3 atm, heating rate of 1 K min^–1^, and temperature range from 278.15 to 393.15 K. Cooling scans are not presented because they are not the focus of this work, but they have always been performed to monitor the thermal reversibility of the micellization processes. The concentration of the triblock copolymer was 0.1% m/m in all experiments to avoid the triblock copolymer-triblock copolymer interaction in the unimeric state at low temperatures.

The raw data from each system were treated with the NanoAnalyze software (TA Instruments) through the following procedure: (i) the baseline obtained in the first experiment was subtracted from the curve obtained in the second experiment to obtain the thermogram associated with each system of triblock copolymer; (ii) each peak in the thermogram resulting from i was adjusted to a fifth-degree polynomial, eliminating all slopes different from those associated with the respective thermal event. Then, the micellization enthalpy change (Δ*H*_mic_) was obtained using eq 4 ^36^

4 $${\Delta H}_{mic}=\int_{T_{1}}^{T_{2}}{\Delta C}_{p}dT$$

where Δ*C*_p_ is the calorific capacity change, at constant pressure, relative to the system in the reference cell, which is expressed by the appropriate polynomial fit as a function of *T*, and *T*_1_ and *T*_2_ are the initial and final temperatures, respectively, of the peak associated with the micellization process induced by temperature increase.

The CMT was defined as the temperature of the peak maximum, located between *T*_1_ and *T*_2_, to decrease the influence of the polydispersity of triblock copolymer on this parameter,^37^ and was determined directly from the maximum of the peak fitted using the NanoAnalyze software. The same procedure was used to obtain the spherical to rod-like micelle transition enthalpy change (Δ*H*_s–r_) and temperature (*T*_s–r_) in the correspondent peaks. The results of the Δ*H* of micellization (or spherical to rod-like transition) were reproducible with a deviation in the peak area of less than 5%, while for CMT (or *T*_s–r_) the deviation was within ±0.5 K.

#### 2.4.2 Isothermal Titration Calorimetry

ITC experiments were performed using a thermal activity monitor (TAM III) controlled by the TAM assistant dedicated software (TA Instruments) and equipped with two removable calorimetric cells (reference and sample) of 4.0 mL each. Experiments were performed at constant temperature (298.15000 ± 0.00001 K) and consisted of the consecutive addition of 25 injections (10 μL each) of 1.7 mmol L^–1^ P123 aqueous solution, using a Hamilton syringe (250 μL) regulated by a 3810 syringe pump, into the sample cell containing initially 2.7 mL of solvent (pure water or water + C_4_mimCl mixture). The same solvent was used to fill the reference cell with the same volume and to prepare the P123 solution inside the syringe. The solution in the sample cell was stirred at 180 rpm (3.0 s^–1^) using a gold helix stirrer during all experiment. The cannula was immersed into the sample cell 120 min before starting the titration. The time interval between two consecutive injections was 600 s, which is time sufficient for the signal to return to the baseline.

### 2.5 Dynamic Light Scattering

Before performing the DLS measurements, all samples were filtered using a 0.22 μm filter and then placed into quartz cuvettes with the aid of a volumetric pipet. The hydrodynamic diameter (*D*_H_) of triblock copolymer micelles in the absence and presence of C_4_mimCl was determined using a Zetasizer Nano-ZS (Malvern Instruments, England), equipped with a He/Ne laser (4 mW, 632.8 nm) and controlled by Zetasizer Nano software. The measurements were performed under a detection angle of 173° with respect to the source, at 306.7 ± 0.1 K. The *D*_H_ of aggregates was calculated using the Stokes–Einstein equation^38^

5 $$D_{H}=\frac{K_{B}T}{3\pi \eta D_{0}}$$

where *K*_B_, *T*, η, and *D*_0_ are the Boltzmann constant, absolute temperature, viscosity of the solvent, and translational diffusion coefficient extrapolated to infinite dilution, respectively.

### 2.6 Data Evaluation and PCA

All data obtained from the different techniques were exported to a Microsoft Excel (2017)-compatible file, where they were manipulated. Posteriorly, the excel data were exported to the OriginLab 2022 software, where all graphs were plotted, and the proper mathematical model fitting was programed and applied when required.

To correlate the effect of the IL on the aggregation thermodynamic parameters (CMT and Δ*H*_mic_) of the triblock copolymer with different structural properties, a PCA was performed with 5% significance, using the XLstat2014 statistical package. The matrices were built using three quantitative variables (IL concentration, MM of copolymer, and PO/EO ratio), two supplementary variables (% CMT and % Δ*H*, which denote the percentages of change in CMT and Δ*H* regarding the system without IL), and a categorical variable (each copolymer). The number of experimental conditions evaluated (defined by the IL concentration, the type of copolymer, and the type of IL) resulted in 76 or 75 observations processed for mixtures containing C_4_mimCl or C_4_mimBr, respectively. The data were normalized and mean-centered to account for the different magnitudes of each variable and to minimize their effect on the PCA distribution. PC1 and PC2 were the two PCs used for making the biplots (loadings and scores), explaining more than 90% of the data, in which each PC is a linear combination of the variables that were investigated in the analysis and has no physical meaning. PC1 is the PC that accounts for the highest variance in the data set, followed by PC2, which captures the maximum variance remaining after PC1, and so forth.

## 3 Results and Discussion

### 3.1 Triblock Copolymer Aggregation Induced by Temperature Increase

A concise overview of the thermodynamic and molecular mechanisms underlying the aggregation behavior of the triblock copolymers in pure water will be presented prior to assessment of the impact of imidazolium-based ILs. Triblock copolymers with the general formula (EO)_*n*_–(PO)_*m*_–(EO)_*n*_, under specific thermodynamic conditions (i.e., concentration and temperature), can self-assemble into micelles, with the (PO)_*m*_ and (EO)_*n*_ blocks forming a hydrophobic core and hydrophilic shell, respectively. This process can be monitored by Nano DSC, a sensitive experimental technique that detects the heat flow associated with different molecular processes induced by temperature increase.^9,39,40^ Figure 1 presents the Nano DSC thermogram for a 0.1% m/m P123 aqueous solution.

**Figure 1:**
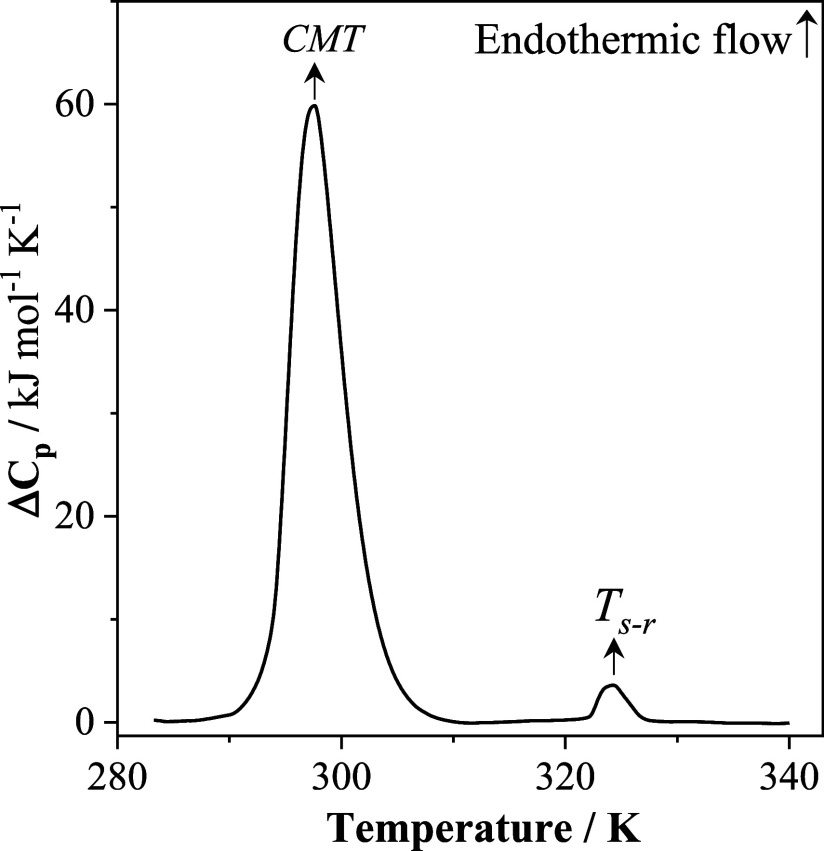
Nano DSC thermogram recorded in the first heating scan of 0.1% m/m P123 aqueous solution, at 3 atm.

As the temperature increases, a pronounced endothermic peak associated with micelle formation appears in the temperature range between 290 and 310 K, indicating that the formation of P123 micelles is entropically driven. The values of CMT and Δ*H*_mic_ obtained were 297.6 K and 405.2 kJ mol^–1^, respectively, and are consistent with those obtained by da Silva et al. (CMT = 298.55 K and Δ*H*_mic_ = 419 kJ mol^–1^).^41^ The second endothermic peak (*T*_s–r_ = 324.9 K and Δ*H*_s–r_ = 10.2 kJ mol^–1^) is associated with the spherical to rod-like micelles transition,^42^ and the values are similar to those obtained by Löf et al.^43^

In pure water, the micellization induced by temperature increase is mainly associated with the difference in the 3D structure of (i) water molecules solvating the PPO hydrophobic segment, more structured and with lower partial molar entropy ( ${\mathrm{S̅}}_{H_{2}O}^{PPO}$ ), and (ii) free water molecules in the bulk, less structured and with higher partial molar entropy ( ${\mathrm{S̅}}_{H_{2}O}^{bulk}$ ). Because of the temperature increase, the difference between the partial molar entropies of those water molecules, i.e., $\Delta \mathrm{S̅}={\mathrm{S̅}}_{H_{2}O}^{bulk}-{\mathrm{S̅}}_{H_{2}O}^{PPO}=f\left(T\right)>0$ , increases, becoming high enough to promote the enthalpic disruption of water-triblock copolymer interactions occurring in the unimeric state at the CMT. Therefore, the P123 aggregation process occurs with the release of water molecules from the solvation shell of the PPO blocks and increase of the entropy of the system. In this process, the positive Δ*H*_mic_ results from a delicate balance of interactions, in which the enthalpic energy required to break the interactions between the water molecules and the PO blocks in the unimeric state is higher than the energy released from the formation of PO–PO interactions in micellar state and water–water interactions in the bulk of the system.^44^

The MM, amount of PO groups, and percentage of EO units in the triblock copolymer can affect its aggregation parameters, such as Δ*H*_mic_ and CMT.^8^ Nano DSC thermograms obtained for L31, L81, L64, and F127 are presented in Figure S2. The obtained Δ*H*_mic_ and CMT parameters are listed in Table 2.

**Table 2:** Thermodynamic Parameters of Aggregation Obtained via Nano DSC for Triblock Copolymers (0.1% m/m) in Pure Water^a^

triblock copolymer	$\frac{{\Delta H}_{mic}}{kJ\;{mol}^{-1}}$	$\frac{CMT}{K}$
L31	42.8	332.6
L81	196.6	304.4
L64	144.7	321.5 (∼303)^9^
P123	405.2 (419)^41^	297.6 (298.55)^41^
F127	289.5 (354)^41^	305.5 (300.84)^41^

a The data reported in parentheses were obtained from literature using DSC. Absence of number in parentheses is related to data not reported in the literature yet.

The CMT increases in the order P123 < L81 ≈ F127 < L64 < L31, indicating that the higher the PO/EO ratio in the triblock copolymer, the lower the thermal stability of the unimers in the solution. Simultaneously, Δ*H*_mic_ increases as the number of PO units increase, that is, L31 < L64 < L81 < F127 < P123 because the higher the number of PO hydrophobic units, the higher the number of water molecules that should be removed from the PO solvation shell,^45^ spending more energy per mole of the triblock copolymer macromolecule. These results suggest that the investigated copolymers provide a suitable gamma of structural and chemical properties that allows the determination of the key role of ILs in modulating their aggregation properties.

#### 3.1.1 Effect of ILs on P123 Aggregation

Nano DSC thermograms of aqueous P123 solutions (0.1% m/m) acquired in the presence of different C_4_mimCl concentrations in the temperature range of 283–342 K are shown in Figure 2.

**Figure 2:**
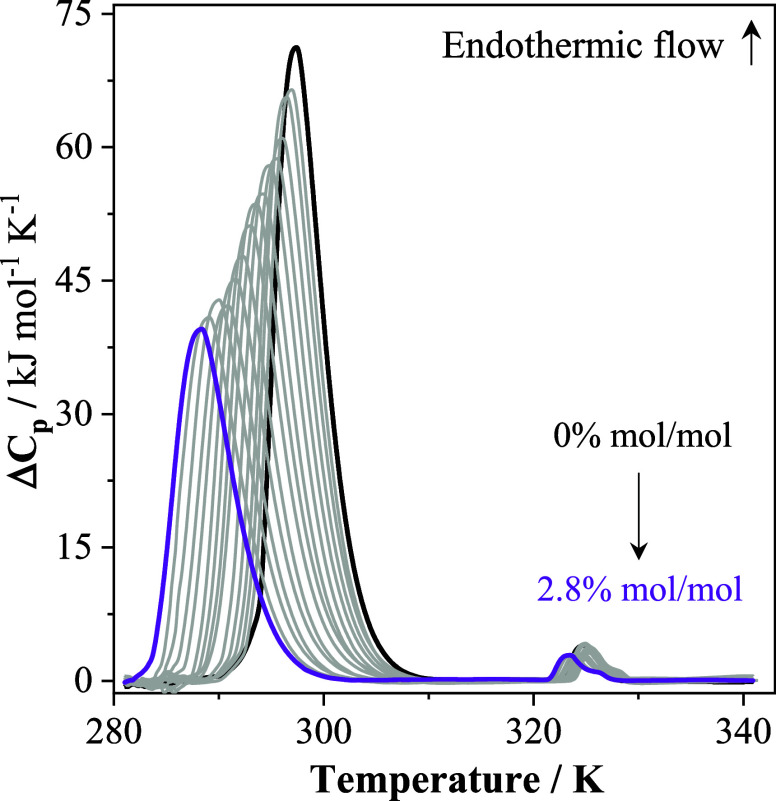
Nano DSC thermograms recorded in the first heating scan of 0.1% m/m aqueous P123 solutions with different C_4_mimCl concentrations at 3 atm.

The increase in the C_4_mimCl concentration promotes a reduction in the first endothermic peak associated with P123 micellization in pure water, which is accompanied by a peak shift to low temperatures. Steady-state fluorescence spectroscopy using pyrene as the fluorescence probe (Figure S3) confirms that the first peak in the thermograms obtained in the presence of the IL is associated with the micellization of P123. Similar results were obtained for C_4_mimBr (Figure S4). The thermograms shown in Figures 2 and S4 were carefully analyzed to obtain the thermodynamic parameters Δ*H*_mic_ and CMT (Table S2), which are plotted against the cosolute concentration in Figure 3. The effect of NaCl on the aggregation of P123 (Figure S5 and Table S3), already evaluated in the literature, was conducted here in the same conditions of study for the ILs to assess the effect of the electrostatic contribution on the P123 micellization process, providing a better understanding of the C_4_mimX effect.^8^ This is supported by the well recognize central position of Na^+^ in the Hofmeister series.^46^

**Figure 3:**
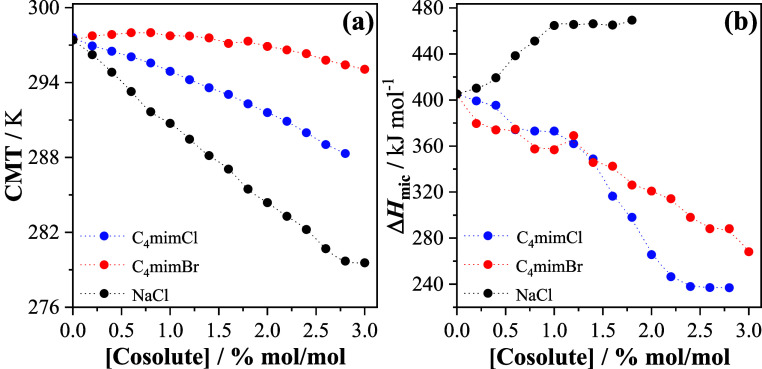
(a) CMT and (b) Δ*H*_mic_ values obtained for P123 micellization in the presence of C_4_mimX (X = Cl^–^ or Br^–^) or aqueous NaCl solutions at different cosolute concentrations. Δ*H*_mic_ values above 2% mol/mol of NaCl were not calculated because the start of the endothermic peak, which is associated with the micellization of P123, was smaller than 280 K (minimum temperature accessed by the equipment).

Similar to the well-established effect of NaCl on triblock copolymer micellization,^8^ an increase in IL concentration up to 2.8% mol/mol decreases the CMT. Moreover, for the same cosolute concentration, the CMT increases in the order NaCl < C_4_mimCl < C_4_mimBr, showing a smaller thermal stabilization of the P123 aggregates in the presence of the IL. Interestingly, this occurs even being the process enthalpically less unfavorable in the presence of the IL (Figure 3b), suggesting that the IL modifies the desolvation process during triblock copolymer micellization in a different way from NaCl.

Regarding the effect of C_4_mimCl and C_4_mimBr, despite the IL ions increasing the configurational entropy of the solvent, which increases the partial molar entropy of the water molecules in the bulk when compared with that of the water molecules solvating the PPO blocks, the C_4_mim^+^ cation is considered a kosmotropic ion that increases the water network structuring in the bulk.^47^ Furthermore, ionic pairs of the IL can replace the water molecules in the PPO solvation shell, displacing some water molecules to the bulk, which decreases the solvophobic effect.^22,48^ Therefore, in the presence of the IL, the entropic gain due to water molecules being released from the PPO solvation shell to the bulk during the micellization process should be smaller than that observed in pure water, thereby stabilizing the unimeric state of the triblock copolymer and promoting micellization at higher CMT values.

To determine the different subprocesses that contribute to the magnitude of Δ*H*_mic_ in the presence of the IL, it is mandatory to determine the location of the IL ionic pairs in the system after the aggregation process, i.e., in the bulk or forming the hydrophobic core of the micelle. To obtain this information, the *D*_H_ of the triblock copolymer micelles was determined via DLS. Figure S6 shows the *D*_H_ values for the P123 aggregates versus C_4_mimCl concentration, obtained from the volume size distribution curves, at 306.7 K.

In pure water, the P123 micelles have a *D*_H_ of 16.3 ± 0.2 nm, close to the value previously reported by Adhikari et al. (18.2 nm).^24^ The increase in the IL concentration from 0 to 3.0% mol/mol promotes the growth of almost 1.8 times the size of the aggregate, which is remarkable for colloidal systems. This result suggests that the IL was incorporated into the P123 micelles. Experiments performed with steady-state fluorescence spectroscopy, monitoring the intrinsic fluorescence of the IL cation, corroborate these results (Figures S7 and S8 and Table S4; and related discussion in the Supporting Information).

Once the IL can solvate the PPO blocks and be transferred to the inner triblock copolymer micelles, Δ*H*_mic_ in the presence of the IL can be considered as a contribution of the subprocesses shown in eq 6. It should be noted, however, that the additive enthalpy model used here is qualitative and assumes independence between the different subprocesses occurring during the micellization process. In complex self-assembly systems such as this, the model should be interpreted as approximate and indicative rather than definitive.^49^

6 $${\Delta H}_{mic}=\Delta H_{w-TC}^{s}+\Delta H_{w-w}^{s}+\Delta H_{w-w}^{b}+{\Delta H}_{TC-TC}^{micelle}+\Delta H_{PPO-IL}^{des}$$

Here, Δ*H*_w–TC_^s^ is the enthalpy change associated with the disruption of water-triblock copolymer interactions in the solvation shell of the triblock copolymer; Δ*H*_w–w_^s^ and Δ*H*_w–w_^b^ are the enthalpy changes associated with the disruption and formation of water–water interactions in the solvation shell of the triblock copolymer and in the bulk of the solution, respectively; and ΔH_TC–TC_^micelle^ is the enthalpy change associated with the formation of triblock copolymer-triblock copolymer interactions in the aggregate, which is mainly due to the formation of PO–PO interactions.

The disruption of water-triblock copolymer interactions and water–water interactions in the solvation shell of the triblock copolymer spends energy and, then, Δ*H*_w–w_^s^ > 0 and Δ*H*_w–TC_^s^ > 0.^44^ The formation of van der Waals interactions in the aggregate and water–water interactions in the bulk releases energy, making Δ*H*_TC–TC_^micelle^ < 0 and Δ*H*_w–w_^b^ < 0.^17^ Therefore, Δ*H*_mic_ of P123 in pure water is positive because |Δ*H*_w–w_^s^ + Δ*H*_w–TC_^s^| > |Δ*H*_TC–TC_^micelle^ + Δ*H*_w–w_^b^|. In the presence of C_4_mimCl, the magnitude of these contributions can be changed, particularly Δ*H*_w–TC_^s^ and Δ*H*_w–w_^s^, since C_4_mimCl can alter the solvation shell of the P123. Then, the contribution Δ*H*_PPO–IL_^des^ appears; it corresponds to the energy change associated with the desolvation of PPO blocks that interact with the ionic pair of C_4_mimCl. Removing water molecules from free PO segments is more energetically unfavorable than removal from IL–PO segments, as water–water interactions on the hydrophobic surface are weakened in the latter. Thus, C_4_mimCl reduces PPO–water contact through preferential solvation of PPO, thereby lowering the energy associated with the desolvation processes, which explains the decrease of Δ*H*_mic_ in the presence of the ILs.

When Cl^–^ is replaced with the Br^–^, an anion with a smaller charge density and hydration radio, a thermal stabilization of the aggregates of P123 in the presence of the IL occurs only at concentrations higher than 1.4% mol/mol, even though C_4_mimBr decreases Δ*H*_mic_ for all concentrations tested (Figure 3). This result is surprising because it does not reflect the higher chaotropic character of Br^–^. However, when compared with C_4_mimCl, Δ*H*_mic_ values in the presence of C_4_mimBr are more endothermic at concentrations above 1.4% mol/mol, indicating that the anion of the IL has an important effect on the solvation of the PPO blocks by the IL. It is likely that the smaller capacity of C_4_mimBr to stabilize the aggregates of P123 is due to its lower ability to solvate the PPO blocks. Logically, this lower ability affects Δ*H*_mic_, especially at higher concentrations where Δ*H*_mic_ becomes more positive in the presence of C_4_mimBr when compared with C_4_mimCl. For low concentrations of the cosolutes, when hydrophobic solvation is less intense, Δ*H*_mic_ shows minimal variation between IL types.

When comparing the effect of the concentration of a classical salt such as NaCl, where mainly electrostatic interactions are present, with that of an imidazolium-based salt, it becomes evident that the weak noncovalent interactions mediated by the imidazolium ring, distinct from purely electrostatic ones, reveal the high potential of these salts to exert thermodynamic control over the aggregation of triblock copolymers.

The thermodynamic approach discussed in this section fills a gap in the energetics of triblock copolymer aggregation in imidazolium-based IL aqueous solutions. Moreover, our discussion supports the data already reported in literature^22,25–27^ and expands the explanation given for each effect, which, without a thermodynamic study, lacks scientific support.

#### 3.1.2 PCA and General Effect of IL–Water Mixtures on the Thermodynamics of Triblock Copolymer Micellization

As discussed in the previous section, aqueous solutions of ILs strongly alter the micellization thermodynamic parameters of the P123 copolymer. To understand the structural properties (e.g., MM, PPO block length, and hydrophilic/hydrophobic balance) of the triblock copolymer, which determine the energetics of the aggregation of any (EO)_*n*_–(PO)_*m*_–(EO)_*n*_ copolymer in C_4_mimX–water mixtures, the aggregation parameters obtained for the other copolymers in Table 1 were obtained in the presence of the IL and are presented in Figures S9 and S10 and Tables S5 and S6. The study of these copolymers provides valuable information about the main structural properties of the triblock copolymers that determine the influence of ILs on aggregation. A comparison between different pairs of compounds provides a unique property that changes from one triblock copolymer to another, such as (i) L31 and L81 or L64 and P123 (having a similar hydrophilic percentage but different MM) and (ii) L81 and L64 (having a similar MM but different hydrophilic/hydrophobic balance).

To compare the effect of the ILs on the CMT and Δ*H*_mic_ of the copolymers, the values of these properties were converted, using eq S2, to the percentage of variation of the property in relation to its value obtained in pure water (Figures S11 and S12). Using these percentages, a PCA was carried out.^50,51^ Figure 4 shows a biplot of the first two PCs of the triblock copolymer + water + C_4_mimX systems.

**Figure 4:**
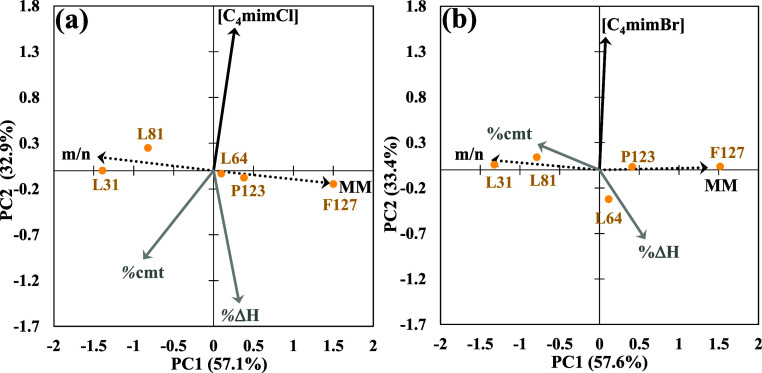
Biplots of the first two PCA components for the data obtained with (a) C_4_mimCl (PC1 + PC2 = 90%) and (b) C_4_mimBr (PC1 + PC2 = 91%). The centroids (●) represent the triblock copolymers and the vectors represent the variables m/n (PO/EO ratio), MM, [C_4_mimX] (IL concentration), % cmt (variation percentage of the CMT), and % Δ*H* (variation percentage of the Δ*H*_mic_).

In both biplots in Figure 4, the distribution of the centroids around the $\vec{MM}$ and $\vec{m/n}$ vectors is observed. The L31 and F127 copolymers, as indicated by the heads of the vectors, show higher PO/EO ratios and MM, respectively. Both $\vec{MM}$ and $\vec{m/n}$ form approximately 90° with $\vec{\left[C_{4}mimX\right]}$ , indicating that these variables are independent of the IL concentration. However, $\vec{\left[C_{4}mimX\right]}$ forms angles ≠ 90° with $\vec{\%\;cmt}$ and $\vec{\%\;\Delta H}$ , suggesting a correlation and dependence among these variables. For C_4_mimCl (Figure 4a), $\vec{\left[C_{4}mimCl\right]}$ has an angle >90° with $\vec{\%\;cmt}$ and $\vec{\%\;\Delta H}$ , implying that the increase in IL concentration in the system promotes further decreases in CMT and Δ*H*_mic_. Although Δ*H*_mic_ shows a similar behavior in the presence of C_4_mimBr (Figure 4b), the CMT value increases as the IL concentration increases (angle < 90°).

By projecting an imaginary line that passes through each centroid and perpendicularly cuts the vectors of each analyzed parameter, it is possible to obtain qualitative information about the values of each property that determine the changes in the response variable in relation to the global mean (origin of PC1 versus PC2 coordinates) (Table S7). Table S8 was constructed using this procedure.

Taking the IL concentration as a reference (Table S8), it can be inferred that by substituting less than 1.4% water molecules for the IL in the solvent, significant changes in the thermodynamic parameters are observed. Under these conditions, copolymers with lower hydrophilic percentages (L31 and L81) exhibit lower Δ*H*_mic_ values than the mean, while CMT increases. An opposite effect on the thermodynamic parameters for the P123, L64, and F127 copolymers is observed.

The main structural difference between these two groups of copolymers is the hydrophilic percentage, which is ≥30% for P123, L64, and F127 and 10% for L81 and L31. This qualitative analysis extracted from PCA suggests that the hydrophilic percentage is the principal structural factor required to generate tendency predictions of CMT and Δ*H*_mic_ of (EO)_*n*_–(PO)_*m*_–(EO)_*n*_ copolymers in aqueous solutions of C_4_mimCl and C_4_mimBr. The multivariate analysis technique, PCA, has emerged as an interesting proposal for the analysis of the aggregation processes of triblock copolymers in the presence of other cosolutes or cosolvents.

#### 3.1.3 Effect of Cosolutes on the Thermodynamics of the Spherical to Rod-Like Micelles Transition

Spherical to rod-like micelle transition is a phenomenon commonly observed in aqueous solutions of triblock copolymers. In such systems, this complex process occurs in two stages: (i) intermicellar interactions and adhesive collisions, and (ii) micellar growth leading to the formation of rod-like micelles.^52–55^ The thermodynamic parameters associated with this phenomenon can provide important information on the role of the cosolutes investigated in this study on the solvation of PEO segments.

As shown in Figure 1, in the range from 320 to 328 K, a second endothermic peak (Δ*H*_s–r_ = 10.2 kJ mol^–1^) is observed in the P123 thermogram associated with the spherical to rod-like micelles, with *T*_s–r_ = 324.9 K. The *T*_s–r_ and Δ*H*_s–r_ for P123 in the presence of different concentrations of IL and NaCl (Tables S2 and S3) are presented in Figure 5.

**Figure 5:**
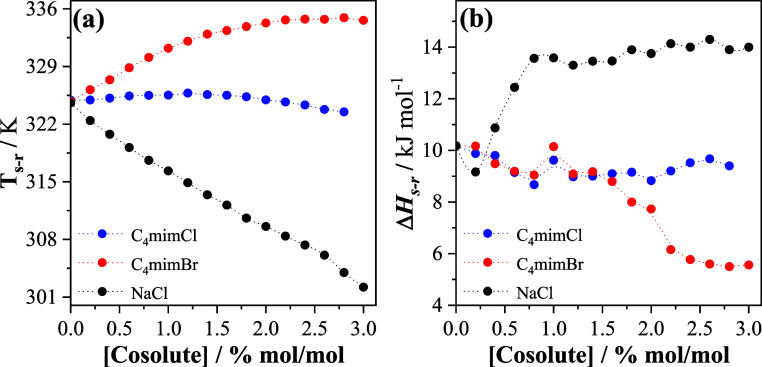
(a) *T*_s–r_ and (b) Δ*H*_s–r_ values obtained for P123 in (i) water + C_4_mimX (X = Cl^–^ or Br^–^) and (ii) water + NaCl mixtures, at different cosolute concentrations.

In the presence of NaCl, the increase in the cosolute concentration causes a decrease in *T*_s–r_ and an increase in Δ*H*_s–r_, with a similar profile to that observed for the respective thermodynamic properties (CMT and Δ*H*_mic_) associated with the micellization processes (Figure 3). However, the profiles of the *T*_s–r_ and Δ*H*_s–r_ versus IL concentration curves were different from those observed in Figure 3. The distinct effects of each cosolute on the parameters of micellization and aggregate shape change indicate that the cosolutes perform a different role in the process driven by PPO blocks desolvation (micellization) when compared with that involving PEO blocks (corona intermicelle interactions).

Although the presence of C_4_mimCl strongly affects P123 micellization, its effect on the micelle shape transition is small. The highest IL concentration only decreases both *T*_s–r_ and Δ*H*_s–r_ by approximately 1.4 units, which can be attributed to its low ability to change the solvation shell of the hydrophilic PEO blocks that form the micelles’ corona. This effect is very distinct when compared with that of NaCl, supporting the hypothesis that, unlike C_4_mim^+^, Na^+^ can bind to PEO segments.^56–58^ Such binding increases the energy associated with electrostatic repulsion between the positively charged micellar coronas (thus increasing Δ*H*_s–r_), while the reduced number of water molecules solvating the PEO blocks in the presence of Na^+^ cations, decreasing *T*_s–r_. Logically, the plateau observed in Δ*H*_s–r_ versus NaCl concentration (but not observed in *T*_s–r_ versus NaCl concentration) suggests saturation of PEO segments by Na^+^ above 1.0% mol/mol.

Interestingly, for C_4_mimBr concentrations of up to 1.4% mol/mol, Δ*H*_s–r_ remains almost constant, and above this concentration, a pronounced decrease in Δ*H*_s–r_ is observed. Despite the process becoming less endothermic, a micelle shape transition occurs at higher *T*_s–r_ as the C_4_mimBr concentration increases. The difference between this result and that obtained with C_4_mimCl provides evidence that both the partitioning of the IL between the bulk and the micelles, as well as the hydration of the PEO segments, are significantly affected by the anion type.

As discussed in Section 3.1.1, the lower ability of C_4_mimBr to form ionic pairs makes its concentration in the inner regions of the triblock copolymer micelles, specifically at the interface between the core and crown of the micelles, smaller than that of C_4_mimCl. Therefore, C_4_mim^+^ and Br^–^ ions probably concentrate relatively more in the bulk of the solution. As a poorly hydrated ion compared with Cl^–^,^59^ Br^–^ increases the amount of water available to interact with the PEO blocks in the corona, which naturally increases the temperature at which the micelle shape transition occurs. Understanding the decrease in Δ*H*_s–r_ is more complex since the shape transition involves a delicate balance of interactions, not only between the interacting molecular species but also due to changes in the aggregate’s surface area and surface composition. In this sense, this phenomenon should be further investigated using other techniques.

This study is the first to report, via DSC, the effects of NaCl and ILs on *T*_s–r_ and Δ*H*_s–r_ of Pluronics, with results consistent with previous findings obtained by other techniques. Increasing NaCl concentration induces a linear decrease in *T*_s–r_, and at ≥1 mol L^–1^, *T*_s–r_ can be reached at room temperature for P85 and P123.^60,61^ McCauley et al.^62^ showed that the anion effect (NaCl vs NaF) on *T*_s–r_ is more pronounced in copolymers with higher MM and PEO fraction, with NaF being more effective due to its preferential interaction with PEO, while NaCl can also partitioning into the PPO/PEO interface. Additionally, dehydration of the micellar corona of P85 has been reported in the presence of organic anions such as salicylate, which promote a sphere-to-rod micellar transition at room temperature.^63^ This phenomenon has been attributed to the incorporation of salicylate into the micellar corona.

### 3.2 Micellization Induced by Concentration Increase

From a thermodynamic perspective, we have discussed triblock copolymer aggregation induced by temperature increase, in which the entropic difference $\Delta \mathrm{S̅}={\mathrm{S̅}}_{H_{2}O}^{bulk}-{\mathrm{S̅}}_{H_{2}O}^{PPO}>0$ needed to overcome the enthalpic barrier of triblock copolymer desolvation is attained at a certain temperature (CMT) because $\frac{{d\mathrm{S̅}}_{H_{2}O}^{bulk}}{dT}>\frac{{d\mathrm{S̅}}_{H_{2}O}^{PPO}}{dT}$ . When the micellization of triblock copolymers occurs because of the concentration increase at a constant temperature, the entropic driving force that promotes aggregation comes from the cumulative effect of adding triblock copolymer unimers in the aqueous solution, which decreases the degrees of freedom of the water molecules in the system due to PPO solvation. Since CMC is a parameter obtained at constant temperature, the effect of temperature on the magnitude of the intermolecular interactions determining the enthalpy changes of micellization is absent, and the effect of cosolutes on the CMC can be distinct from those affecting the CMT. Figure 6a shows the dependence of the CMC of P123 on the cosolute concentration at 298.2 K.

**Figure 6:**
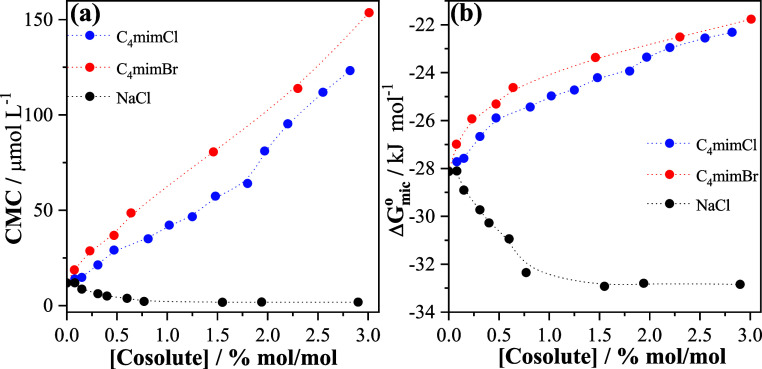
Values of (a) CMC and (b) Δ*G*_mic_^o^ for the P123 triblock copolymer as a function of cosolute concentration at 298.2 K. The data were obtained from Figures S13–S16 and reported in Table S9.

The increase in the concentration of both ILs in the system increases the CMC, with higher values obtained in the presence of C_4_mimBr. In the presence of NaCl, the opposite effect is observed, in which the CMC decreases slightly as the NaCl concentration increases, remaining almost constant for cosolute concentrations higher than 1.5% mol/mol. These results indicate that the C_4_mim^+^ cation increases the solubility of the unimers of the triblock copolymer in solution, while Na^+^ cations favor the formation of micelles. Interestingly, while the IL does not favor the aggregation of the triblock copolymer induced by increases in concentration, an opposite result was observed in the aggregation induced by the temperature increase (Figure 3a). However, in the presence of NaCl, stabilization of the micelles is observed for both processes, confirming that the IL interacts with the triblock copolymer through hydrophobic solvation.

To evaluate the thermodynamic stability of the P123 micelles in the presence of the cosolutes, Δ*G*_mic_^o^ values were calculated using eq 3 and are plotted in Figure 6b (and listed in Table S9). For the concentration range studied, Δ*G*_mic_^o^ is negative, indicating that, at equilibrium, micelles predominate over the unimeric state in the system. Δ*G*_mic_^o^ values become less negative in the presence of the IL, whereas the opposite effect is caused by NaCl, suggesting that the formation of micelles in the system is destabilized by imidazolium cations but stabilized by sodium cations.

Similar to the processes induced by increases in temperature, ITC measurements (Figure S17) suggested that the P123 aggregation process becomes less endothermic with an increasing C_4_mimCl concentration. This result indicates that in the process induced by increases in concentration, the decrease in the entropy changes of micellization in the presence of the IL, as discussed in Section 3.1.1, is the reason for the CMC increase. This inversion of the driving force determining the effect of the IL on triblock copolymer aggregation for temperature or concentration increases is probably associated with the temperature at which both processes occur. During aggregation induced by concentration increases, the temperature (298.2 K) is always higher than the onset temperature (i.e., the temperature at the beginning of the endothermic peak in the nano DSC thermogram, see Figure 2). For the same IL concentration, the decrease in the positive entropic change term dominates the effect of IL on the stabilization of the micelles.

## 4 Conclusions

Calorimetric, spectrophotometric, and statistical approaches were used to evaluate the effect of ILs (or NaCl) on the thermodynamic parameters for the aggregation of the (EO)_*n*_–(PO)_*m*_–(EO)_*n*_ triblock copolymers. New insights into the energetics of triblock copolymer aggregation in IL aqueous solutions have emerged, contributing to the understanding of the effect of these electrolytes on the aggregation thermodynamics of triblock copolymers.

The thermodynamic parameters of the aggregation strongly depended on the cations and anions of the cosolute. The effect of the IL on triblock copolymer micellization also depended on the process that induced aggregation, that is, increasing the concentration or temperature. The micellization thermodynamic parameters of P123 were strongly affected by the IL concentration. The CMT increased in the order NaCl < C_4_mimCl < C_4_mimBr, and the aggregation process became less endothermic in the presence of C_4_mimX (Cl^–^ > Br^–^) but more endothermic in NaCl solutions.

PCA showed that the EO percentage in the triblock copolymers was the main structural aspect of the copolymers that determined the effect of the IL on triblock copolymer aggregation. When the effects of the IL on the energetic parameters were evaluated, opposite effects were observed for triblock copolymers with lower (10%) and higher (≥30%) percentages of EO. For the micellization induced by increases in concentration, the effect of the IL on the stability of P123 micelles was the opposite of that observed for the micellization induced increase in temperature, whereas for NaCl, the tendencies were the same in both processes.

We suggest that different effects are caused by the classical and IL salts; Na^+^ interacts with the PEO blocks of the triblock copolymer, while C_4_mim^+^, forming an ionic pair with the counterion, preferentially solvates the PPO block. The capacity of the IL anion to form ionic pairs with the imidazolium cation, associated with its kosmotropic ability, also affects the dehydration processes of the hydrophobic segments of PPO, micellization, and the hydrophilic blocks of PEO in the spherical to rod-like micelle transition. Future work would benefit from in-depth calorimetric investigations to explore the role of cation hydrophobicity and the effects of more complex anions in ILs.

## Supplementary Material



## Notes

Molecular structure of the IL cation; nano DSC thermograms of triblock copolymer in cosolute + water mixtures; quotient of pyrene vibrational band intensities (*I*_1_/*I*_3_) vs log­([P123]/μmol L^–1^) obtained in different water + electrolyte mixtures; hydrodynamic diameter and volume size distribution for P123 in C_4_mimCl + water mixtures; normalized emission intensity spectra of C_4_mimCl in water + triblock copolymer mixtures; percentage of C_4_mimCl species distribution in water or water + triblock copolymer mixture; variation percentage of thermodynamic properties as a function of IL concentration; quotient of pyrene vibrational band intensities (*I*_1_/*I*_3_) versus log­([P123]/% m/m) obtained in pure water and water + cosolute mixtures; Δ*H*_obs_ as a function of [P123] in water + C_4_mimCl solution; overview of key publications investigating the aggregation of triblock copolymers in binary systems consisting of water and imidazolium-based ILs; thermodynamic parameters of triblock copolymer aggregation (Δ*H*_mic_, CMT, Δ*H*_tr_, *T*_tr_, CMC, and Δ*G*_mic_^o^); spectroscopic parameters (*l*_ex_, *l*_em_, *I*, ϕ_sp_) of C_4_mimCl in P123 micellar environments; descriptive statistical data obtained through PCA; quantification of IL species in spectrofluorometric measurements; variation percentage of the measured thermodynamic property for each IL concentration, in relation to its value obtained in pure water; and description and discussion of complementary results (PDF)
