# Supplementary material for: Modulation of Solvent Properties Using Imidazolium-Based Ionic Liquids: Effects on the Thermodynamics of PEO–PPO–PEO Triblock Copolymer Aggregation
Source: ACS Omega. 2025 Sep 29;10(40):47481–92. doi: 10.1021/acsomega.5c07226 (PMC12529128; doi:10.1021/acsomega.5c07226)
Supplement: Supplementary file 1 [file ao5c07226_si_001.pdf]

Modulation of solvent properties using imidazolium-based ionic liquids: Effects on the thermodynamics of PEO-PPO-PEO triblock copolymer aggregation

Álvaro Javier Patiño-Agudelo,[1,2] Guilherme Max Dias Ferreira,[1,3] Gabriel Max Dias Ferreira,[1,4] Yara Luiza Coelho,[1,5] Isabela A. Marques,[1] Jaqueline P. Rezende,[6,7] Ana Clarissa dos Santos Pires,[7] and Luis Henrique Mendes da Silva*[1]

[1]*Advanced Thermokinetics of Molecular Systems (ATOMS) Group, Chemistry Department, Federal University of Viçosa, Viçosa, Av. P. H. Rolfs s/s, 36570900, Viçosa-MG, Brazil.*

[2]*Department of Physical Chemistry, Institute of Chemistry, University of Campinas, UNICAMP, Campinas 13083-970, São Paulo, Brazil*

[3]*Group of Material, Interface, and Solutions (MatIS), Department of Chemistry, Universidade Federal de Lavras, Campus Universitário, 37200000, Lavras-MG, Brazil.*

[4]*Department of Chemistry, Federal University of Ouro Preto, Campus Universitário, 35400000, Ouro Preto-MG, Brazil.*

[5]*Chemistry Institute, Federal University of Alfenas, Campus Universitário, 37130000, Alfenas-MG, Brazil.*

[6]*Department of Food Science, Federal University of Lavras, Campus Universitário, 37200000, Lavras-MG, Brazil.*

[7]*Applied Molecular Thermodynamics (THERMA), Department of Food Technology, Federal University of Viçosa, Av. P. H. Rolfs s/s, 36570900, Viçosa-MG, Brazil.*

E-mails:
patinoagudeloaj@gmail.com
guilherme.ferreira@ufla.br
gabriel.ferreira@ufop.edu.br
yaracoelhof@gmail.com
marques.isabela100@gmail.com
jaquelinerezende@ufla.br
ana.pires@ufv.br

**Corresponding author:* luhen@ufv.br; Phone: +55 31 36126633; Fax: +55 31 38992175

Table of contents

1. Introduction 3

3. Results and discussion 4

3.1 Triblock copolymer aggregation induced by increases in temperature 4

3.1.1 Effect of ILs on P123 aggregation 5

Micellization of P123 in IL+H_2O mixtures using pyrene methodology 5

DSC data of P123 obtained in water + IL mixtures 7

DSC data of P123 obtained in water + NaCl mixtures...8

Dynamic light scattering data 9

Intrinsic fluorescence of C_4mim^+ in presence and absence of P123 micelles......10

3.1.2 Principal components analysis (PCA) and general effect of IL-water mixtures on the thermodynamics of triblock copolymer micellization 13

3.2 Micellization induced by increases in concentration 18

Determination of the CMC of P123 in pure water and electrolyte + water mixtures using pyrene methodology 18

Isothermal titration calorimetry (ITC) results 22

References 24

1. Introduction

Table S1. Overview of key publications investigating the aggregation of triblock copolymers in binary systems consisting of water and imidazolium-based ionic liquids (ILs). Reported properties include critical micelle temperature (CMT), critical micelle concentration (CMC), cloud point (CP), hydrodynamic radius (R_H), and standard enthalpy (ΔH^0_{mic}), entropy (ΔS^0_{mic}), and free energy (ΔG^0_{mic}) changes of micellization. Arrows pointing down or up represent decreases or increases in physical properties, respectively

Year	Triblock copolymer	Ionic liquid	CMT	CMC	CP	R_H	ΔG^0_{mic}	ΔH^0_{mic}	ΔS^0_{mic}	Ref
2007	P104	C_4mimBr	↓	-	-	↑	-	-	-	1
2008	P123	C_4mimBr	-	-	-	↑	-	-	-	2
2009		C_4mimBF$_4$	-	-	-	↓	-	-	-	3
2012	P103	C_4mimBF$_4$	-	-	↑	↑	-	-	-	4
		C_6mimBF$_4$	-	-	↑	↑	-	-	-	
		C_8mimBF$_4$	-	-	↓	↓	-	-	-	
2014	F108	C_4mimX[a]	↓	-	-	-	-	-	-	5
2016		AmimCl	↓	-	-	↑	-	-	-	6
		BzmimCl	↓	-	-	↑	-	-	-	
2017		$C_n$mimCl*	↓	-	-	-	-	-	-	7
	F127	C_8mimX[b]	-	↑	-	↓	↑	-	-	8
		$C_n$mimCl**	-	↑	-	↓	↑	-	-	
2018	P123	C_4mimBF$_4$	-	↑	-	-	↑	↑	↑	9,10
2022	F127	$C_n$mimBr***	-	-	-	↓	-	-	-	10
	L62	C_4mimCl	↓	-	-	-	-	-	-	11

[a]: X = HSO_4^-, CH_3COO^-, Cl^-, I^-, BF_4^- or SCN^-.
[b]: X = Cl^-, Br^-, or I.
*: n = 2, 4, 6 or 10.
**: n = 4, 6 or 8.
***: n = 8, 10, 12 or 14.

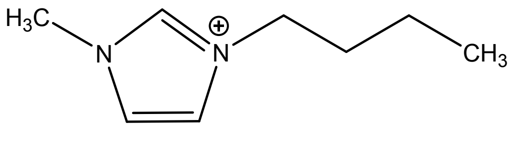

Figure S1. 1-butyl-3-methylimidazolium cation.

3. Results and discussion

3.1 Triblock copolymer aggregation induced by increases in temperature

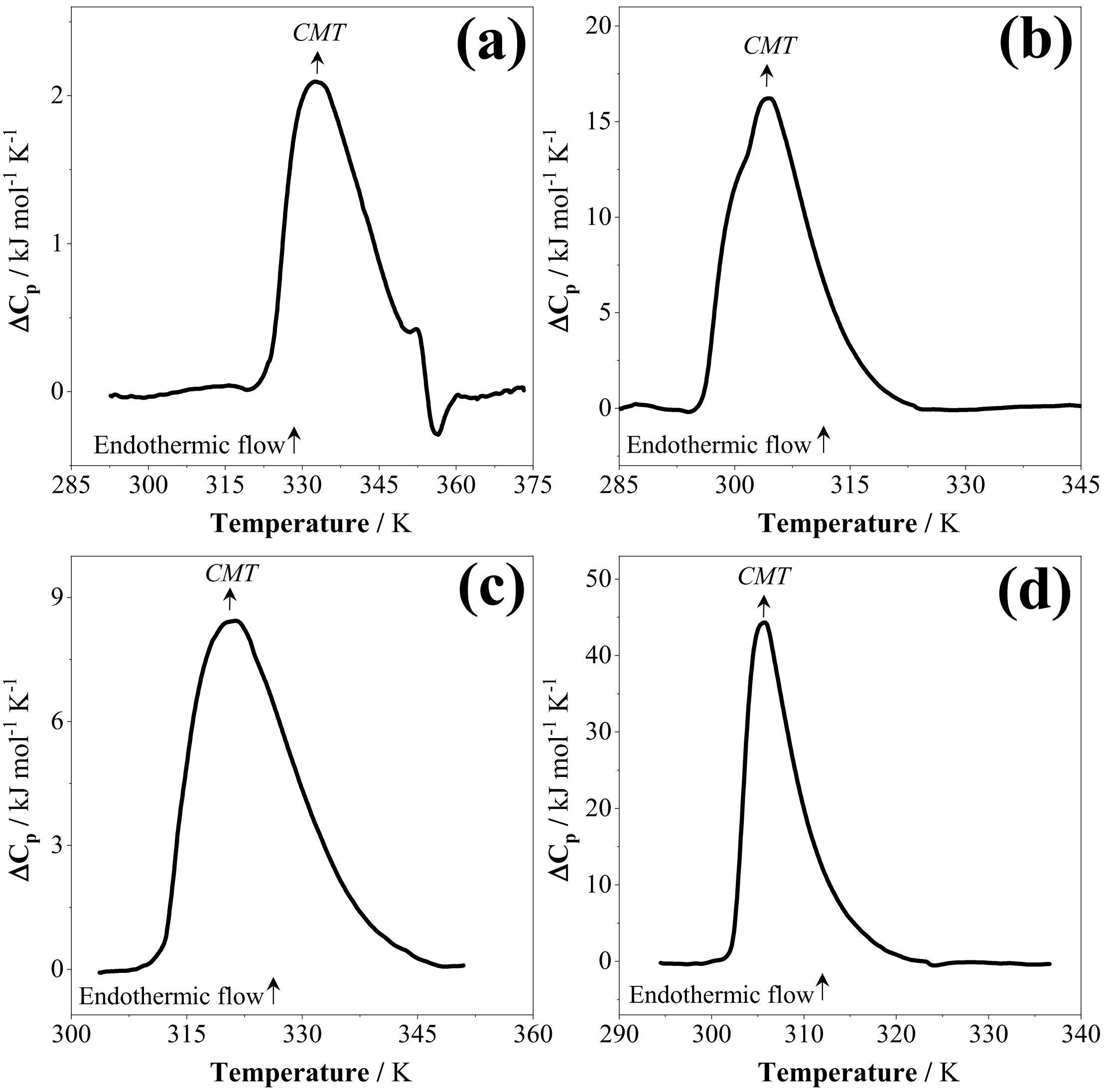

Figure S2. Nano DSC thermograms recorded during the first heating scan of aqueous solutions of triblock copolymer (0.1% m/m), at 3 atm. **(a)** L31, **(b)** L81, **(c)** L64, and **(d)** F127.

3.1.1 Effect of ILs on P123 aggregation

Micellization of P123 in IL+H_2O mixtures using pyrene methodology

Steady-state fluorescence spectroscopy, using pyrene as the fluorescence probe, is a strategic technique for indicating the formation of surfactant aggregates since the I_1/I_3 ratio in the pyrene spectra is sensitive to the microenvironment where the pyrene is located in the system. For instance, when pyrene is solvated by pure water, the value of I_1/I_3 is approximately 1.8, while in less polar environments, such as the interior of a micelle, this ratio is around 1.1.[12] In this sense, fluorescence spectra of aqueous solutions containing P123 (0.1% m/m) + C_4mimCl were obtained at the temperatures of 283.2 and 308.2 K, for different IL concentrations (Figure S3). The two temperatures selected correspond to temperatures in which a base line is observed in the Nano DSC thermograms for all IL concentrations, after and before the first endothermic peak.

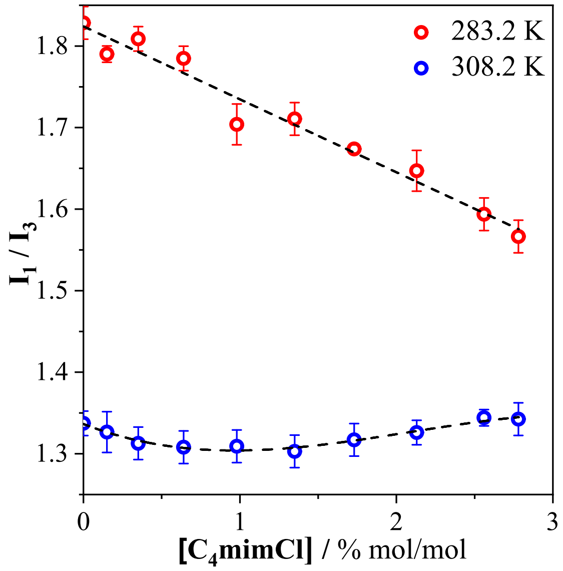

Figure S3. Quotient of pyrene vibrational band intensities (I_1/I_3) *versus* [C_4mimCl] curves obtained for P123 (0.1% m/m) + C_4mimCl (0 < [C_4mimCl] ≤ 2.8 % mol/mol) mixtures, at 283.2 and 308.2 K. The lines (−) represent the linear (I_1/I_3= 1.824 - 0.984[C_4mimCl], r^2= 0.999) and third-degree polynomial (I_1/I_3= 1.336 - 0.074[C_4mimCl] + 0.05[C_4mimCl]^2 – 0.008[C_4mimCl]^3, r^2= 0.999) fitting of the data obtained at 283.2 and 308.2 K, respectively.

At 283.2 K, the I_1/I_3 values decrease linearly from 1.83, in pure water, to 1.6 when the C_4mimCl concentration increased from 0 to 2.8% mol/mol. This is evidence that P123 molecules are in unimeric state and the pyrene solvation layer is gradually altered with the substitution of water molecules by IL, which decreases the polarity of the probe's microenvironment. At 308.2 K, the I_1/I_3 values follow a polynomial behavior with values of 1.3, in pure water, and 1.34, at 2.8% mol/mol C_4mimCl. These values indicate that the pyrene molecules are preferentially located in a more hydrophobic environment, *i.e.*, the core of the P123 micelles.

DSC data of P123 obtained in water + IL mixtures

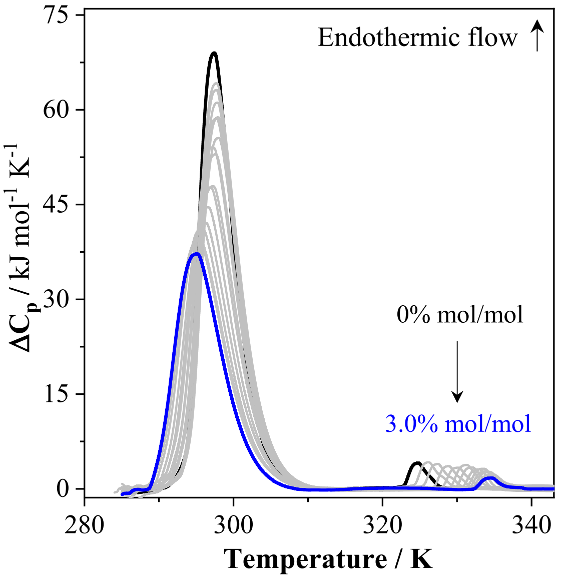

Figure S4. Nano DSC thermograms recorded in the first heating scan of 0.1% m/m P123 aqueous solutions with different C_4mimBr concentrations, at 3 atm.

Table S2. Thermodynamic parameters (ΔH_{mic}, CMT, ΔH_{s-r}, and T_{s-r}) of the P123 (0.1% m/m) aggregation process, obtained at different IL concentrations, using nano DSC.

[IL]	C_4mimBr				C_4mimCl			
	ΔH_{mic}	CMT	ΔH_{s-r}	T_{s-r}	ΔH_{mic}	CMT	ΔH_{s-r}	T_{s-r}
0	405.2	297.6	10.2	324.9	405.2	297.6	10.2	324.9
0.2	379.6	297.7	10.2	326.2	399.2	296.9	9.9	324.9
0.4	374.0	297.9	9.5	327.4	395.4	296.5	9.8	325.2
0.6	374.6	298.0	9.2	328.9	374.2	296.1	9.2	325.4
0.8	357.5	298.0	9.0	330.1	373.1	295.6	8.7	325.5
1.0	356.8	297.8	10.2	331.2	373.0	294.9	9.6	325.5
1.2	369.0	297.7	9.1	332.1	362.0	294.2	9.0	325.8
1.4	345.7	297.6	9.2	332.9	348.7	293.6	9.0	325.6
1.6	342.5	297.1	8.8	333.4	316.4	293.1	9.1	325.5
1.8	326.1	297.3	8.0	333.9	298.1	292.3	9.2	325.3
2.0	320.9	296.9	7.7	334.3	265.7	291.6	8.8	324.9
2.2	314.3	296.6	6.2	334.7	246.5	290.9	9.2	324.7
2.4	298.1	296.3	5.8	334.8	238.0	290.0	9.5	324.3
2.6	288.3	295.8	5.6	334.7	237.1	289.0	9.7	323.8
2.8	288.2	295.4	5.5	334.9	236.9	288.3	9.4	323.5
3.0	268.2	295.1	5.6	334.6				

The units of [IL], CMT (or T_{s-r}) and ΔH_{mic} (or ΔH_{s-r}) are % mol/mol, K and kJ mol^{-1}, respectively.

DSC data of P123 obtained in water + NaCl mixtures

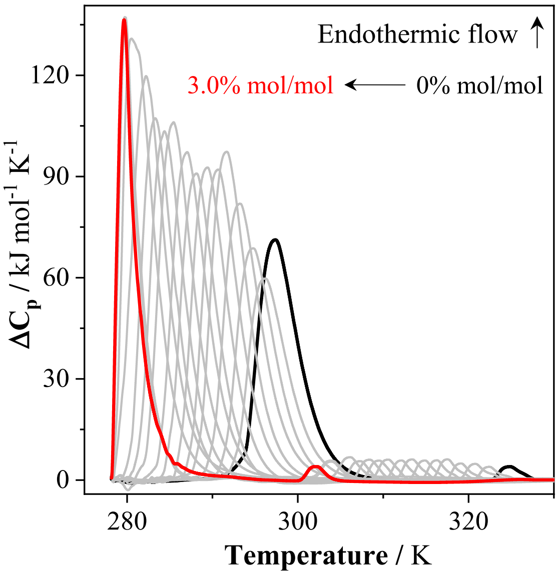

Figure S5. Nano DSC thermograms recorded in the first heating scan of 0.1% m/m P123 aqueous solutions in different NaCl concentrations, at 3 atm.

Table S3. Thermodynamic parameters (ΔH_{mic}, CMT, ΔH_{s-r}, and T_{s-r}) of the P123 aggregation process, obtained for 0.1% m/m triblock copolymer aqueous solutions at different NaCl concentrations, using nano DSC. ΔH_{mic} values above 2% mol/mol of NaCl were not calculated because the start of the endothermic peak, which is associated with the micellization of P123, was smaller than 280 K.

[NaCl]	P123			
	ΔH_{mic}	CMT	ΔH_{s-r}	T_{s-r}
0	405.2	297.6	10.2	324.9
0.2	410.2	296.2	9.2	322.4
0.4	419.3	294.8	10.9	320.8
0.6	438.5	293.3	12.4	319.2
0.8	451.2	291.7	13.6	317.6
1.0	464.7	290.7	13.3	316.3
1.2	465.6	289.5	13.5	314.9
1.4	466.2	288.2	13.5	313.4
1.6	465.1	287.1	13.5	312.2
1.8	469.3	285.5	13.9	310.6
2.0	-	284.4	13.8	309.6
2.2	-	283.3	14.1	308.4
2.4	-	282.2	14.0	307.3
2.6	-	280.7	14.3	306.1
2.8	-	279.7	13.9	304.0
3.0	-	279.6	14.0	302.2

The units of [NaCl], CMT (or T_{s-r}), and ΔH_{mic} (or ΔH_{s-r}) are % mol/mol, K, and kJ mol^{-1}, respectively.

Dynamic light scattering data

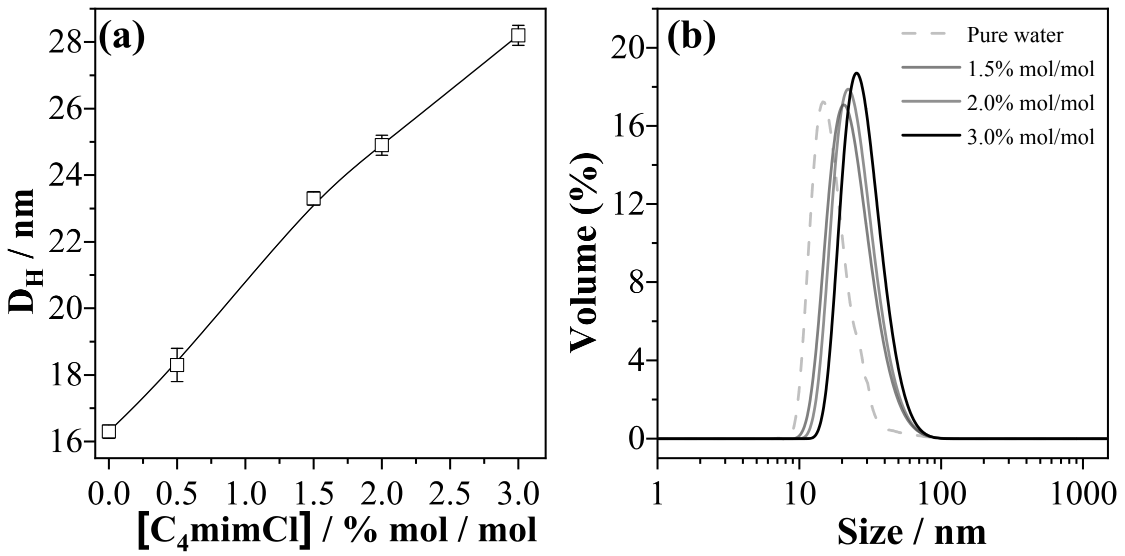

Figure S6. (a) Values of D_H and **(b)** volume size distribution for 0.1% m/m P123 aqueous solution in different C_4mimCl concentrations, at 306.7 K (experiment temperature was 9 K above the CMT obtained in pure water through DSC experiments).

Intrinsic fluorescence of C_4mim^+ in presence and absence of P123 micelles

An alternative for identifying the IL species present inside the P123 micelles is by monitoring spectroscopically the C_4mim^+ ions in solution. Paul *et al.*,[13] in 2005, showed the C_4mim^+ cation is characterized by absorption in the entire UV region, and the excitation wavelength-dependent shift of the fluorescence maximum is attributed to the existence of different IL chemical species (supramolecules with different stoichiometry and structure) in an aqueous solution. In this way, steady-state fluorescence spectroscopy experiments were carried out with C_4mimCl 3.0% mol/mol aqueous solutions, in the absence and presence of P123 0.1% m/m, using different excitation wavelengths (Figure S7), at 298.2 K.

For each emission spectrum, the intensity associated with the peak maximum was collected. Assuming that the intensity of emission is only a function of the concentration of each species in the system, the fraction of these species can be quantified using equation S1.

$$\phi_{sp\,(i)} = \frac{I_{l\,(i)}}{\sum I_{l\,(i)}} x100 \qquad \text{S1}$$

where $\phi_{sp\,(i)}$ is the percentage of species i, and $I_{l\,(i)}$ is the maximum intensity. All C_4mimCl species were quantified (Table S4) and plotted against the excitation wavelength (Figure S8).

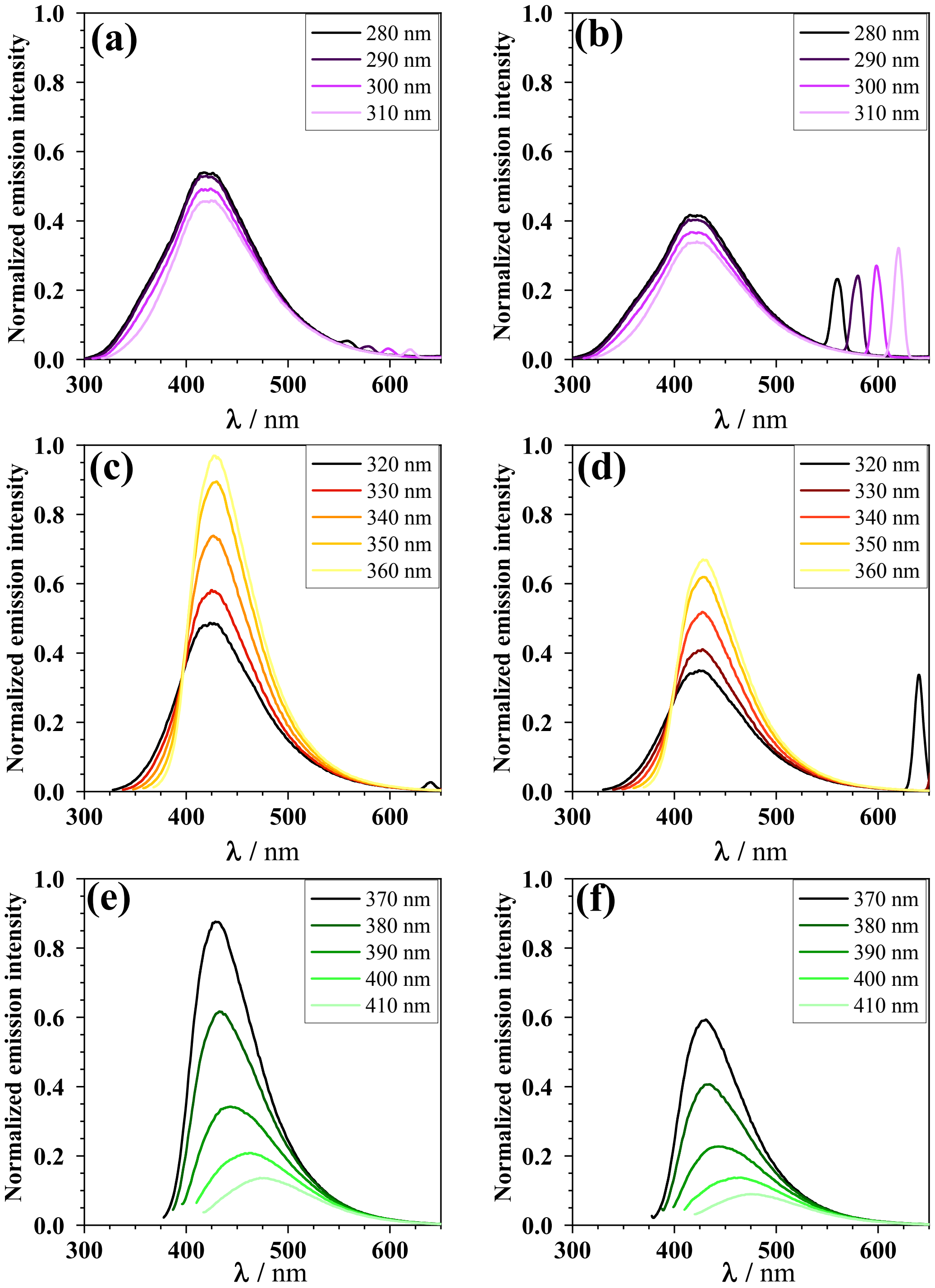

Figure S7. Normalized emission intensity spectra of C_4mimCl (3.0% mol/mol) using different wavelengths of excitations (inset) in absence [**(a)**, **(c)** and **(e)**] and presence [**(b)**, **(d)** and **(f)**] of 0.1% m/m P123, at 298.2 K.

Table S4. Wavelength of excitation (l_{ex}), wavelength of maximum emission (l_{em}), maximum of intensity (I), and percentage of species distribution (ϕ_{sp}) of C_4mimCl (3.0% mol/mol) in pure water or 0.1% m/m P123 aqueous solution.

l_{ex}	C_4mimCl + water			C_4mimCl + P123 + water		
nm	l_{em} / nm	I (a.u)	% ϕ_{sp}	l_{em} / nm	I (a.u)	% ϕ_{sp}
280	424.5	521.3 ± 0.2	6.8	424.2	404.8 ± 0.7	7.5
290	424.5	510.7 ± 0.3	6.7	423.9	394.7 ± 0.7	7.4
300	426.1	478.0 ± 0.3	6.3	425.3	362.5 ± 0.8	6.7
310	429.0	448.9 ± 0.3	5.9	427.9	338 ± 1	6.3
320	431.7	475.4 ± 0.4	6.2	430.8	347 ± 1	6.4
330	433.9	562.5 ± 0.4	7.4	433.8	395.8 ± 0.4	7.3
340	435.2	715.9 ± 0.4	9.4	435.1	499.6 ± 0.4	9.3
350	436.1	872.6 ± 0.4	11.4	436.0	602.9 ± 0.4	11.2
360	437.1	945.8 ± 0.5	12.4	437.0	650.9 ± 0.5	12.1
370	439.2	850.5 ± 0.5	11.1	439.2	572.9 ± 0.5	10.6
380	444.2	589.1 ± 0.5	7.7	444.1	387.7 ± 0.5	7.2
390	454.7	333.4 ± 0.5	4.4	454.2	220.2 ± 0.5	4.1
400	467.0	202.2 ± 0.4	2.6	466.9	133.4 ± 0.3	2.5
410	481.5	132.3 ± 0.3	1.7	481.2	87.0 ± 0.3	1.6

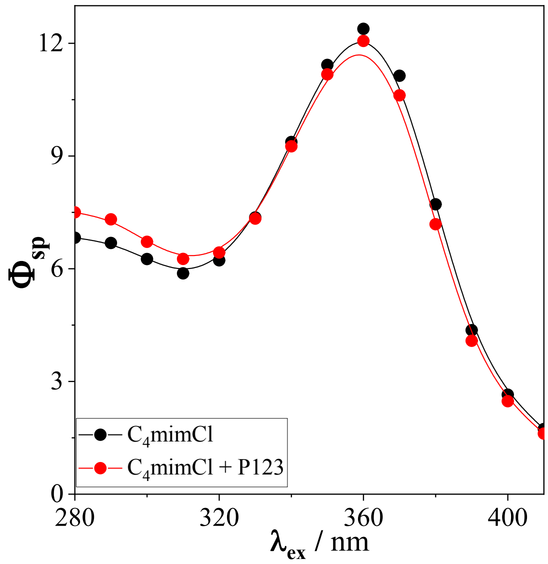

Figure S8. Percentage of C_4mimCl (3.0% mol/mol) species distribution in pure water and in P123 aqueous solution (0.1% m/m), at 298.2 K (temperature approximately 10 K above the CMT obtained using DSC).

The presence of P123 copolymer changes the distribution percentage of C_4mimCl species, increasing the concentration of species associated with excitation wavelengths < 320 nm and decreasing the ones associated with wavelengths > 340 nm. This increase in species with shorter excitation wavelengths may be associated with a partition of these species into the micelles, therefore shifting the thermodynamic balance of the system.

3.1.2 Principal components analysis (PCA) and general effect of IL-water mixtures on the thermodynamics of triblock copolymer micellization

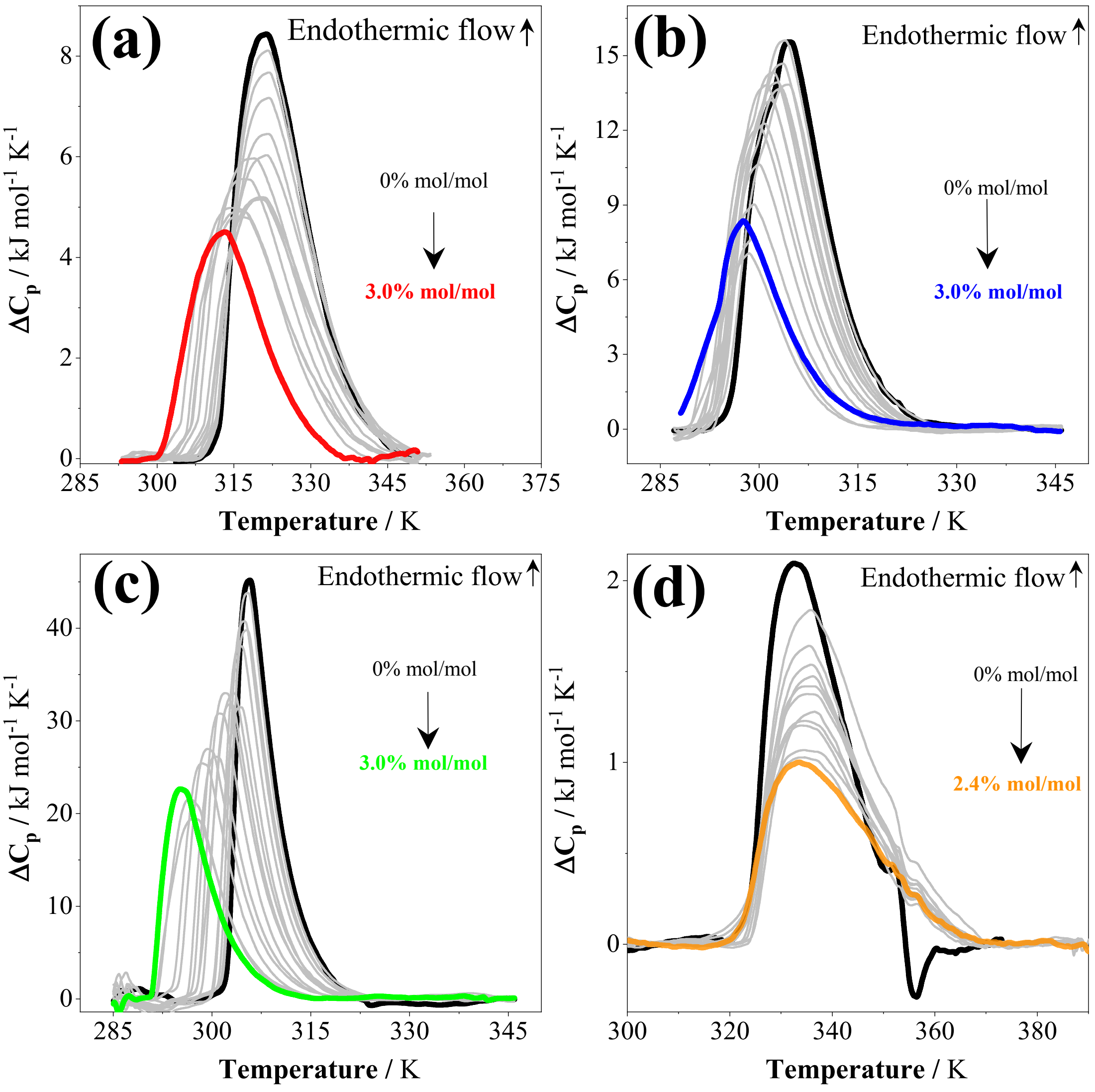

Figure S9. Nano DSC thermograms recorded in the first heating scan of 0.1% m/m aqueous solutions of **(a)** L64, **(b)** L81, **(c)** F127, and **(d)** L31 with different C_4mimCl concentrations, at 3 atm.

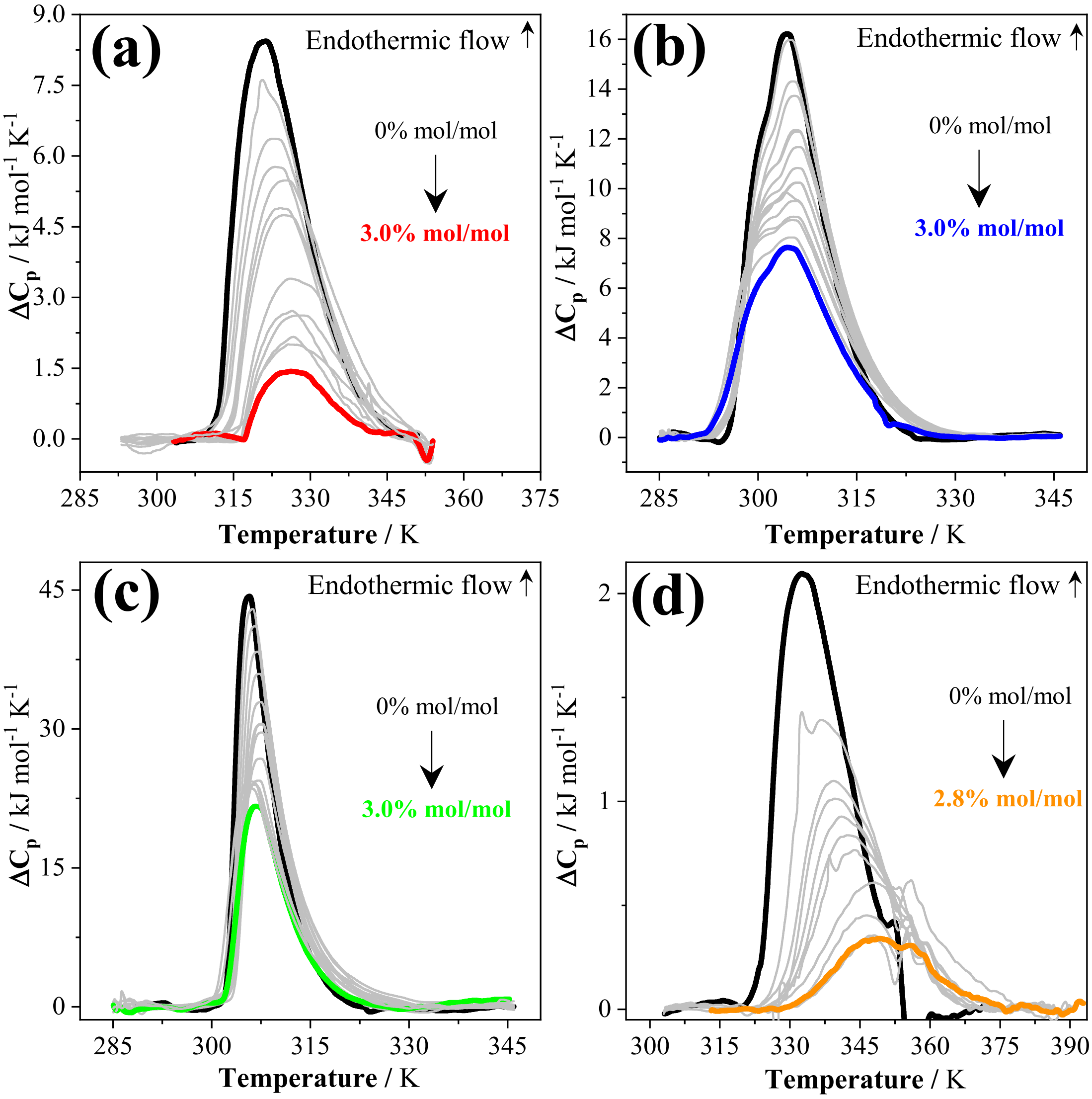

Figure S10. Nano DSC thermograms recorded in the first heating scan of 0.1% m/m aqueous solutions of **(a)** L64, **(b)** L81, **(c)** F127, and **(d)** L31 with different C_4mimBr concentrations, at 3 atm.

Table S5. Thermodynamic parameters (ΔH_{mic} and CMT) of the triblock copolymer (L64 and L81) (0.1% m/m) aggregation process, obtained at different IL concentrations, using nano DSC.

[IL]	L64				L81			
	C_4mimCl		C_4mimBr		C_4mimCl		C_4mimBr	
	ΔH_{mic}	CMT	ΔH_{mic}	CMT	ΔH_{mic}	CMT	ΔH_{mic}	CMT
0	144.7	321.5	144.7	321.5	196.6	304.4	196.6	304.4
0.2	141.8	321.7	107.9	320.6	188.7	304.5	208.1	304.9
0.4	135.6	321.8	99.8	322.4	186.6	303.9	207.0	305.2
0.6	132.0	321.9	90.7	323.4	186.6	303.7	203.8	305.5
0.8	129.8	321.7	87.0	323.8	177.3	303.3	199.0	305.7
1.0	127.0	321.6	79.8	325.3	167.2	302.7	193.5	305.8
1.2	124.5	321.4	71.8	326.3	163.0	302.2	190.2	306.1
1.4	115.8	321.1	56.0	326.0	150.9	301.8	173.1	305.9
1.6	108.5	320.6	43.3	326.5	146.5	301.1	157.7	305.9
1.8	105.0	320.3	43.0	325.8	134.1	300.7	157.6	305.8
2.0	101.1	319.0	38.9	326.8	132.1	300.1	155.4	305.6
2.2	97.2	318.0	39.2	326.5	122.0	300.0	154.1	305.5
2.4	95.3	317.8	29.3	327.1	123.3	299.2	129.3	305.3
2.6	95.9	317.1	-	-	102.5	298.9	129.0	305.3
2.8	91.6	314.9	-	-	86.2	298.5	123.0	304.8
3.0	-	-	-	-	79.0	297.6	111.1	304.6

The units of [IL], CMT and ΔH_{mic} are % mol/mol, K and kJ mol^{-1}, respectively.

Table S6. Thermodynamic parameters (ΔH_{mic} and CMT) of the triblock copolymer (F127 and L31) (0.1% m/m) aggregation process, obtained at different IL concentrations, using nano DSC.

[IL]	F127				L31			
	C_4mimCl		C_4mimBr		C_4mimCl		C_4mimBr	
	ΔH_{mic}	CMT	ΔH_{mic}	CMT	ΔH_{mic}	CMT	ΔH_{mic}	CMT
0	289.5	305.5	289.5	305.5	42.8	332.6	42.8	332.6
0.2	278.5	305.3	290.4	306.3	38.5	334.2	30.5	336.4
0.4	270.1	305.2	283.5	306.6	38.1	335.7	27.7	338.8
0.6	266.6	304.8	272.2	306.9	34.8	336.0	21.9	339.6
0.8	263.0	304.2	262.1	307.2	31.6	336.2	21.0	339.5
1.0	255.9	304.2	256.0	307.6	28.4	336.6	16.0	341.0
1.2	251.9	303.4	254.8	307.7	27.8	336.4	15.0	342.3
1.4	247.7	302.7	252.6	307.4	25.2	336.4	13.7	343.4
1.6	248.5	301.9	249.3	307.4	22.3	335.5	11.5	343.9
1.8	241.2	301.1	234.4	307.2	21.3	335.2	5.1	346.6
2.0	212.4	300.5	220.0	307.2	19.4	334.6	8.8	346.5
2.2	195.2	299.3	204.7	307.2	19.3	334.5	5.4	349.0
2.4	191.5	298.4	200.6	306.7	19.3	333.6	6.3	347.2
2.6	187.6	297.2	200.7	306.1	-	-	7.9	348.1
2.8	191.5	296.5	197.8	305.5	-	-	4.8	350.8
3.0	186.8	295.2	195.4	305.8	-		-	-

The units of [IL], CMT and ΔH_{mic} are % mol/mol, K and kJ mol^{-1}, respectively.

$$\%Y = \frac{Y - Y^*}{Y^*} x100 \qquad \text{S2}$$

$\%Y$ is the variation percentage of the measured property, for each IL concentration, in relation to its value obtained in pure water. Y and Y^* are the property values obtained in a determined IL concentration and pure water, respectively.

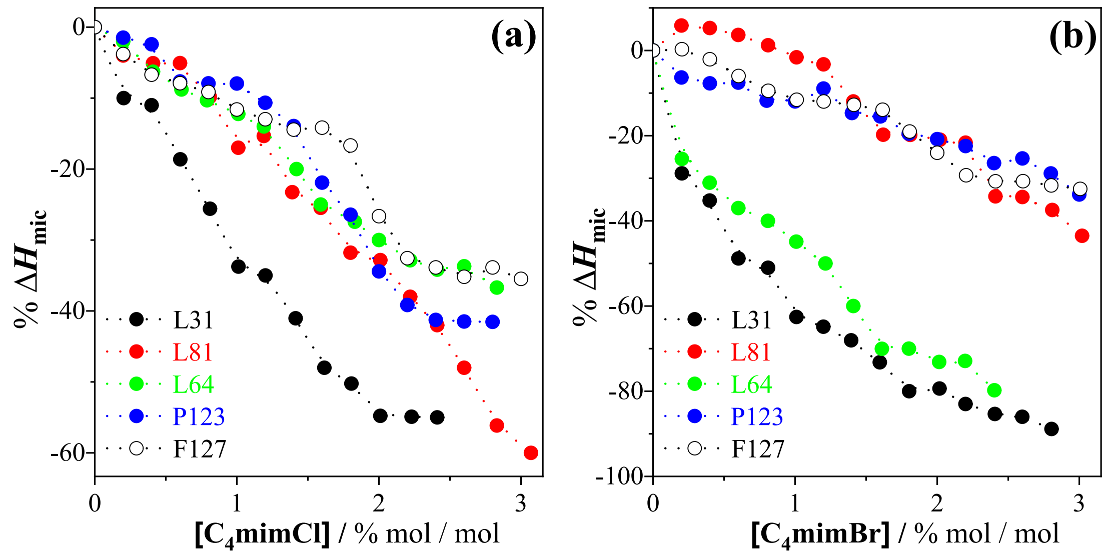

Figure S11. Variation percentage of ΔH_{mic} as a function of **(a)** C_4mimCl and **(b)** C_4mimBr concentration. The concentration of the copolymers was 0.1% m/m.

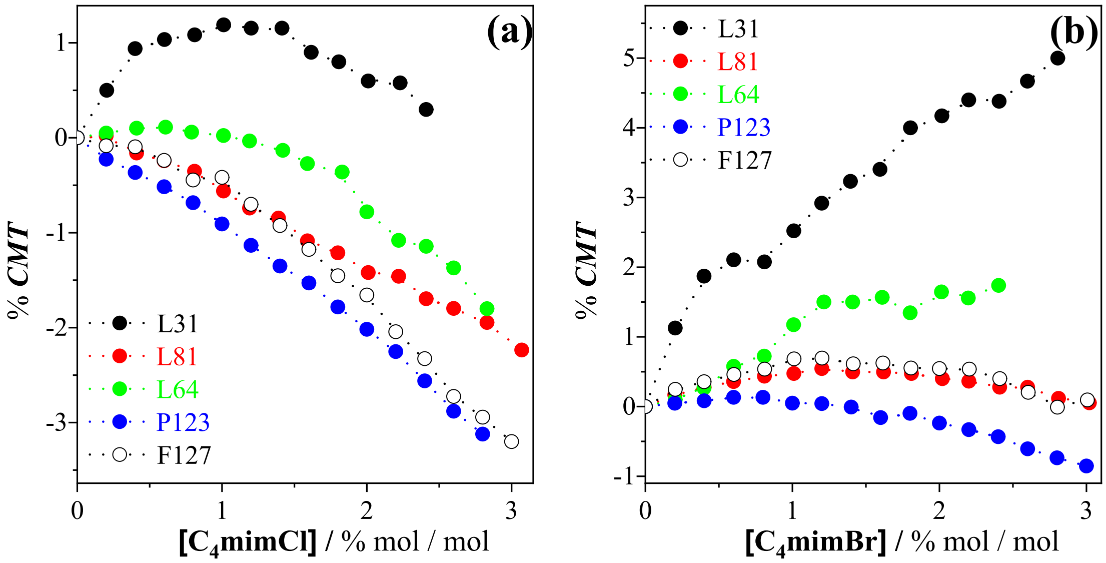

Figure S12. Variation percentage of CMT as a function of **(a)** C_4mimCl and **(b)** C_4mimBr concentration. The concentration of the copolymers was 0.1% m/m.

Table S7. Descriptive statistical data obtained through PCA for the data set determined in the presence of C_4mimCl and C_4mimBr. The values reported for [C_4mimX] (IL concentration), %ΔH (variation percentage of ΔH_{mic}), and $\%CMT$ (variation percentage of CMT) correspond to the mean value of each property and are related to the origin of the PC1 *versus* PC2 coordinates.

Variable	C_4mimCl	C_4mimBr
**[C_4mimX]** / % mol/mol	1.4	1.4
$\%\boldsymbol{\Delta H}$ / kJ/mol	-23	-31
$\%\boldsymbol{CMT}$ / K	-0.7	0.9

Table S8. Descriptive qualitative analysis for each copolymer in the biplot, using the data from Table S7.

Variable	C_4mimCl			C_4mimBr		
	**%ΔH**	$\%\boldsymbol{CMT}$	**[C_4mimCl]**	**%ΔH**	$\%\boldsymbol{CMT}$	**[C_4mimBr]**
**L31**	> -23	< -0.7	< 1.4	> -31	> 0.9	= 1.4
**L81**	> -23	< -0.7	< 1.4	> -31	> 0.9	= 1.4
**L64**	< -23	> -0.7	≈ 1.4	< -31	< 0.9	< 1.4
**P123**	< -23	> -0.7	≈ 1.4	< -31	< 0.9	≈ 1.4
**F127**	< -23	> -0.7	= 1.4	< -31	< 0.9	≈ 1.4

3.2 Micellization induced by increases in concentration

Determination of the CMC of P123 in pure water and electrolyte + water mixtures using pyrene methodology

One of the most important parameters obtained from the micellization of triblock copolymer, at constant temperature, is the critical micelle concentration (CMC). This property indicates the concentration at which the triblock copolymer unimers associate to form a polymolecular aggregate with greater size and fixed aggregation number.[14] In this work, the CMC of P123, in the absence and presence of co-solutes, was determined by monitoring the ratio between the vibrational bands of the pyrene (I_1/I_3) as a function of triblock copolymer concentration using fluorescence spectroscopy, at 298.2 K. Figure S13 present the I_1/I_3 *versus* log([P123] / % m/m) curve for P123 in pure water. Similar curves for the P123 in electrolyte + water mixtures are presented in Figures S14-S16 and the CMC values are listed in Table S9.

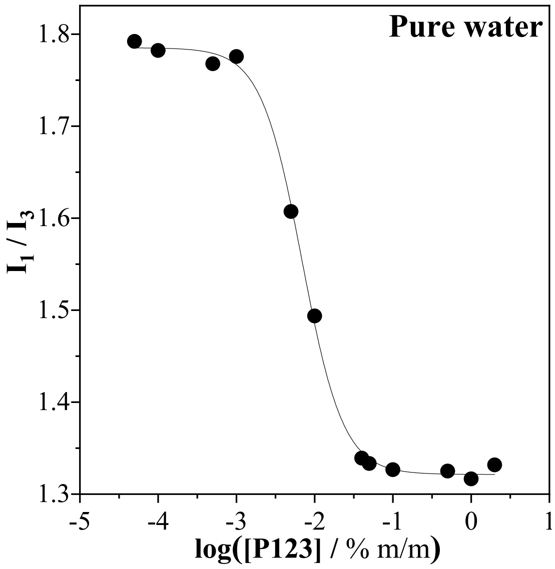

Figure S13. Quotient of pyrene vibrational band intensities (I_1/I_3) *versus* log([P123] / % m/m) obtained in pure water at 298.2 K. (−) Fitting model to determine the CMC.

As can be seen in Figure S13, a classic sigmoidal profile was observed,[15] where the first (-4.3 < log[P123] / % m/m < -3.2; $I_1/I_3 \approx 1.8$) and second (-0.9 < log[P123] / % m/m <

0.3; $I_1/I_3 \approx 1.3$) plateaus indicate the presence of unimers and an unimers-micelles equilibrium, respectively, in the system. Between the plateaus (-3.2 < log[P123] / % m/m < -0.9; $\Delta(I_1/I_3) \approx 0.5$) a transition region is observed, with an inflection point at log[P123] / % m/m= -2.16. The P123 concentration at this inflection point (11.8 μmol L^{-1}) was taken as the CMC of the triblock copolymer and correspond to the concentration of P123 where the enthalpic barrier for the formation of micelles in pure water is surpassed. This value agrees with that reported by Lee *et. al.*.[16]

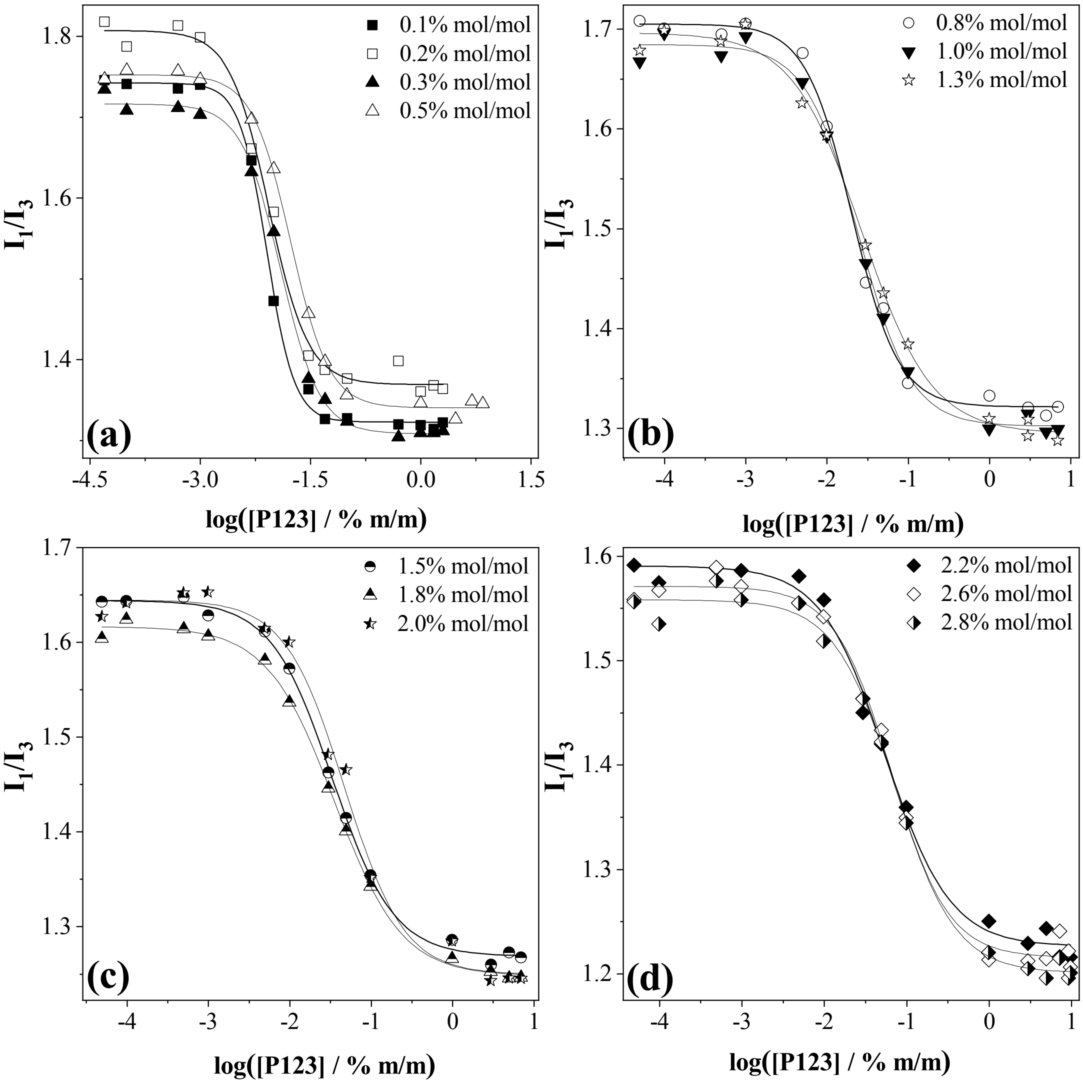

Figure S14. Quotient of pyrene vibrational band intensities (I_1/I_3) *versus* log([P123] / % m/m) obtained in C_4mimCl + water mixtures. **(a)** [C_4mimCl] ≤ 0.5% mol/mol, **(b)** 0.8% mol/mol ≤ [C_4mimCl] ≤ 1.3% mol/mol, **(c)** 1.5% mol/mol ≤ [C_4mimCl] ≤ 2.0% mol/mol, and **(d)** 2.2% mol/mol ≤ [C_4mimCl] ≤ 2.8% mol/mol at 298.2 K. (−) Fitting model to determine the CMC.

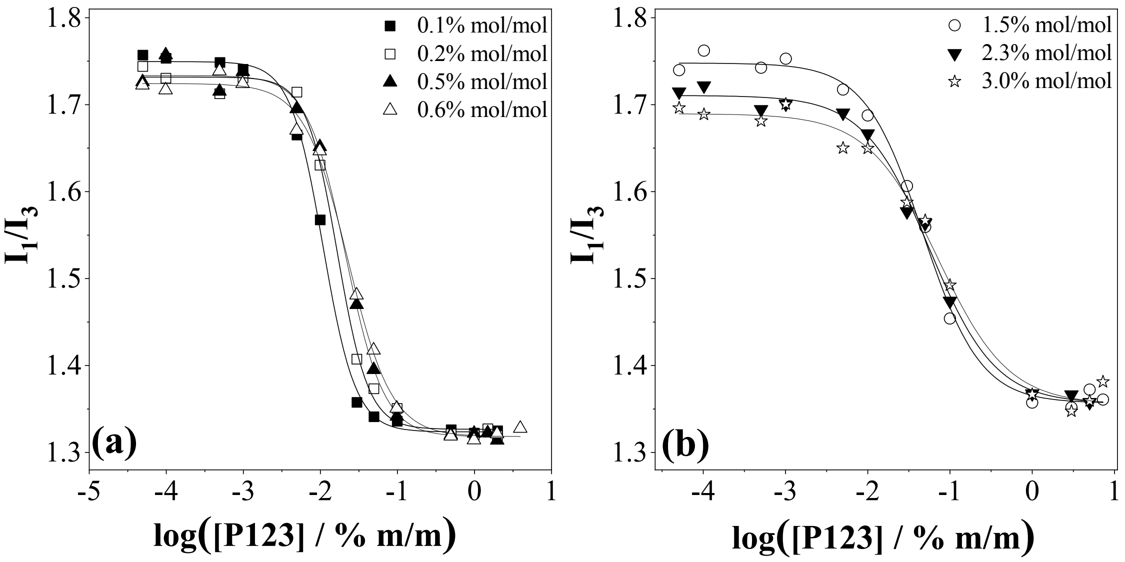

Figure S15. Quotient of pyrene vibrational band intensities (I_1/I_3) *versus* log([P123] / % m/m) obtained in C_4mimBr + water mixtures. **(a)** [C_4mimBr] ≤ 0.6% mol/mol, and **(b)** 1.5% mol/mol ≤ [C_4mimBr] ≤ 3.0% mol/mol at 298.2 K. (−) Fitting model to determine the CMC.

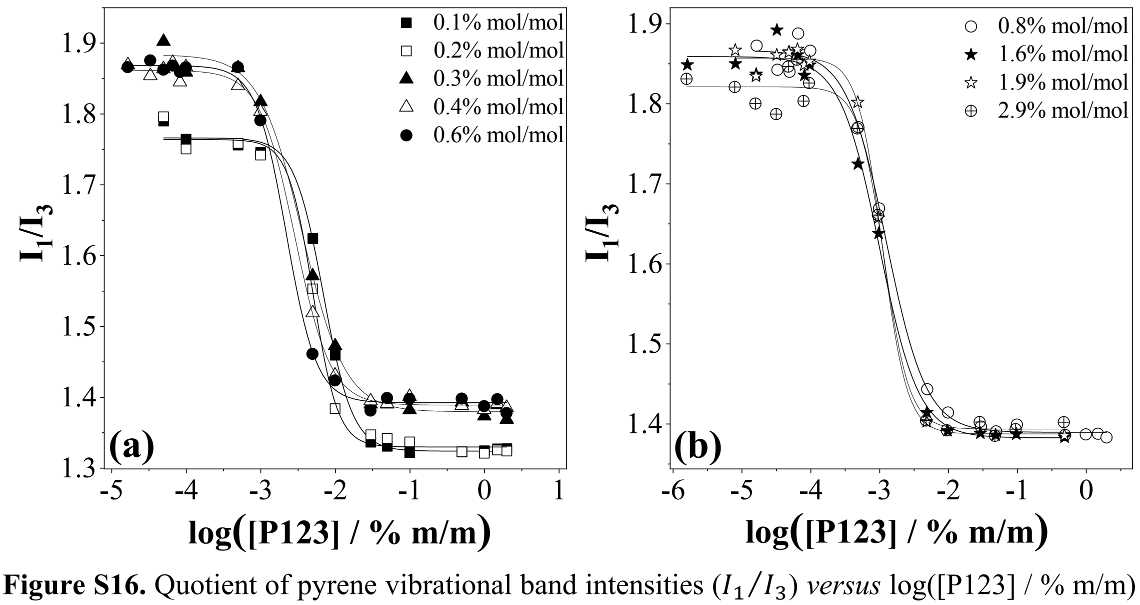

Figure S16. Quotient of pyrene vibrational band intensities (I_1/I_3) *versus* log([P123] / % m/m) obtained in NaCl + water mixtures. **(a)** [NaCl] ≤ 0.6% mol/mol, and **(b)** 0.8% mol/mol ≤ [NaCl] ≤ 2.9% mol/mol at 298.2 K. (−) Fitting model to determine the CMC.

Table S9. Critical micellar concentration (CMC) and standard Gibbs free energy change (ΔG^{o}_{mic}) values obtained for the P123 micellization in different IL + water or NaCl + water mixtures, at 298.2 K.

[C_4mimCl]	CMC	ΔG^{o}_{mic}	[C_4mimBr]	CMC	ΔG^{o}_{mic}	[NaCl]	CMC	ΔG^{o}_{mic}
0	11.8	−28.1	0	11.8	−28.1	0	11.8	−28.1
0.1	13.9	−27.7	0.1	18.7	−27.0	0.1	11.9	−28.1
0.2	14.8	−27.6	0.2	28.7	−25.9	0.2	8.6	−28.9
0.3	21.3	−26.7	0.5	36.8	−25.3	0.3	6.2	−29.7
0.5	29.1	−25.9	0.6	48.6	−24.6	0.4	5.0	−30.3
0.8	35.0	−25.4	1.5	80.6	−23.4	0.6	3.8	−30.9
1.0	42.2	−25.0	2.3	113.9	−22.5	0.8	2.2	−32.3
1.3	46.6	−24.7	3.0	153.7	−21.8	1.6	1.7	−32.9
1.5	57.4	−24.2	-	-	-	1.9	1.8	−32.8
1.8	64.1	−23.9	-	-	-	2.9	1.8	−32.8
2.0	81.0	−23.4	-	-	-	-	-	-
2.2	95.4	−23.0	-	-	-	-	-	-
2.6	111.9	−22.6	-	-	-	-	-	-
2.8	123.3	−22.3	-	-	-	-	-	-

The units of [co-solute], CMC, and ΔG^{o}_{mic} are % mol/mol, μmol L^{-1} and kJ mol^{-1}, respectively. The error associated with the CMC and ΔG^{o}_{mic} values is lower than 1%.

Isothermal titration calorimetry (ITC) results

In order to make a complete analysis of the energetic contributions to the ΔG^{o}_{mic} values, the determination and discussion of the standard micellization enthalpy (ΔH^{o}_{mic}) and entropy (ΔS^{o}_{mic}) changes are essential. ΔH^{o}_{mic} can be determined using the van't Hoff approximation[17] or the isothermal titration calorimetry technique.[18]

Cooperative processes dominated by weak interactions, such as the micellization of copolymers, are multi-step with very close entropy values between different states, making $\Delta H^{o}_{mic,ITC} \neq \Delta H^{o}_{mic,van't\,Hoff}$.[19] Therefore, ITC was used for determining ΔH^{o}_{mic} since the van't Hoff approximation would lead to incorrect conclusions. Due to the absence of a sigmoidal profile in the ITC curves (Figure S17), it was not possible to determine ΔH^{o}_{mic} ($\Delta H^{o}_{mic} = \Delta H_{ob2} - \Delta H_{ob1}$) because the regions of the interactions unimer-unimer (ΔH_{ob1}) and micelle-micelle (ΔH_{ob2}) cannot be distinguished.[20] However, a qualitative analysis of the thermograms obtained can be made.

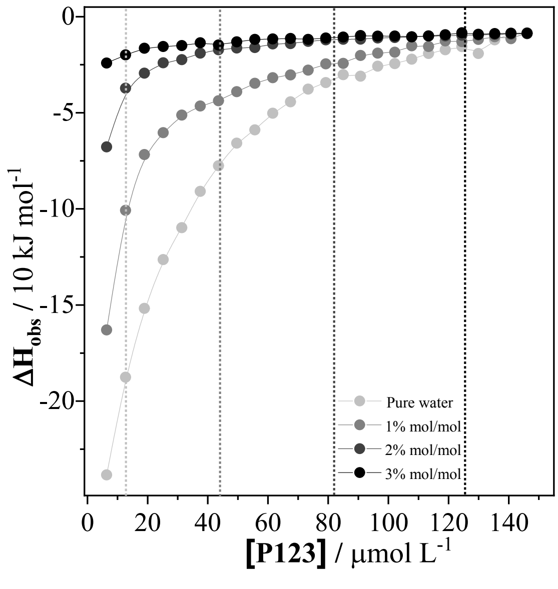

Figure S17. ΔH_{obs} vs. [P123] curves obtained by dilution of P123 solution in C_4mimCl + water mixtures, at 298.15 K. The vertical lines are the CMC values of P123 obtained by pyrene method in each IL concentration.

For all C_4mimCl concentrations, the increase of P123 concentration caused the increase of ΔH_{ob} until a plateau (ΔH_{ob2}), at [P123] > 115 μmol L^{-1}. Moreover, the P123 aggregation process becomes less endothermic with increasing C_4mimCl concentration (result expected from the tendence in the increase of ΔH_{ob1}). From this result, the higher the concentration of C_4mimCl in the system, the greater the amount of IL interacting with the P123 unimers and, consequently, the energy cost for desolvating the unimers decreases.

References

(1) Zheng, L.; Guo, C.; Wang, J.; Liang, X.; Chen, S.; Ma, J.; Yang, B.; Jiang, Y.; Liu, H. Effect of Ionic Liquids on the Aggregation Behavior of PEO-PPO-PEO Block Copolymers in Aqueous Solution. *J Phys Chem B* **2007**, *111* (6), 1327–1333. https://doi.org/10.1021/jp066727c.

(2) Adhikari, A.; Dey, S.; Das, D. K.; Mandal, U.; Ghosh, S.; Bhattacharyya, K. Solvation Dynamics in Ionic Liquid Swollen P123 Triblock Copolymer Micelle: A Femtosecond Excitation Wavelength Dependence Study. *J Phys Chem B* **2008**, *112* (20), 6350–6357. https://doi.org/10.1021/jp7118857.

(3) Dey, S.; Adhikari, A.; Das, D. K.; Sasmal, D. K.; Bhattacharyya, K. Femtosecond Solvation Dynamics in a Micron-Sized Aggregate of an Ionic Liquid and P123 Triblock Copolymer. *J Phys Chem B* **2009**, *113* (4), 959–965. https://doi.org/10.1021/jp804401p.

(4) Parmar, A.; Aswal, V. K.; Bahadur, P. Interaction between the Ionic Liquids 1-Alkyl-3-Methylimidazolium Tetrafluoroborate and Pluronic P103 in Aqueous Solution: A DLS, SANS and NMR Study. *Spectrochim Acta A Mol Biomol Spectrosc* **2012**, *97*, 137–143. https://doi.org/https://doi.org/10.1016/j.saa.2012.05.075.

(5) Madhusudhana Reddy, P.; Venkatesu, P. Influence of Ionic Liquids on the Critical Micellization Temperature of a Tri-Block Co-Polymer in Aqueous Media. *J Colloid Interface Sci* **2014**, *420*, 166–173. https://doi.org/https://doi.org/10.1016/j.jcis.2014.01.006.

(6) Umapathi, R.; Venkatesu, P. Solution Behavior of Triblock Copolymer in the Presence of Ionic Liquids: A Comparative Study of Two Ionic Liquids Possessing Different Cations with Same Anion. *ACS Sustain Chem Eng* **2016**, *4* (4), 2412–2421. https://doi.org/10.1021/acssuschemeng.6b00137.

(7) Umapathi, R.; Venkatesu, P. Thermo-Responsive Triblock Copolymer Phase Transition Behaviour in Imidazolium-Based Ionic Liquids: Role of the Effect of Alkyl Chain Length of Cations. *J Colloid Interface Sci* **2017**, *485*, 183–191. https://doi.org/https://doi.org/10.1016/j.jcis.2016.09.034.

(8) Lunagariya, J.; Kumar, N. S.; Asif, M.; Dhar, A.; Vekariya, R. L. Dependency of Anion and Chain Length of Imidazolium Based Ionic Liquid on Micellization of the Block Copolymer F127 in Aqueous Solution: An Experimental Deep Insight. *Polymers 2017, Vol. 9, Page 285* **2017**, *9* (7), 285. https://doi.org/10.3390/POLYM9070285.

(9) He, Z.; Ma, Y.; Alexandridis, P. Comparison of Ionic Liquid and Salt Effects on the Thermodynamics of Amphiphile Micellization in Water. *Colloids Surf A Physicochem Eng Asp* **2018**, *559*, 159–168. https://doi.org/https://doi.org/10.1016/j.colsurfa.2018.09.061.

(10) Luo, H.; Jiang, K.; Wang, X.; Yao, H.; Liang, X.; Li, Y.; Liu, H. How Multiple Noncovalent Interactions Regulate the Aggregation Behavior of Amphiphilic Triblock Copolymer/Surface-Active Ionic Liquid Mixtures. *J Mol Liq* **2022**, *363*, 119856. https://doi.org/https://doi.org/10.1016/j.molliq.2022.119856.

(11) Heller, W. T.; Do, C. Impact of Two Water-Miscible Ionic Liquids on the Temperature-Dependent Self-Assembly of the (EO)6–(PO)34–(EO)6 Block Copolymer. *ACS Omega* **2022**, *7* (23), 19474–19483. https://doi.org/10.1021/acsomega.2c01166.

(12) Goddard, E. D.; Turro, N. J.; Kuo, P. L.; Ananthapadmanabhan, K. P. Fluorescence Probes for Critical Micelle Concentration Determination. *Langmuir* **1985**, *1* (3), 352–355. https://doi.org/10.1021/la00063a015.

(13) Paul, A.; Mandal, P. K.; Samanta, A. On the Optical Properties of the Imidazolium Ionic Liquids. *J Phys Chem B* **2005**, *109* (18), 9148–9153. https://doi.org/10.1021/jp0503967.

(14) Riess, G. Micellization of Block Copolymers. *Prog Polym Sci* **2003**, *28* (7), 1107–1170. https://doi.org/https://doi.org/10.1016/S0079-6700(03)00015-7.

(15) Leiva, A.; Quina, F. H.; Araneda, E.; Gargallo, L.; Radić, D. New Three-Arm Amphiphilic and Biodegradable Block Copolymers Composed of Poly(ε-Caprolactone) and Poly(N-Vinyl-2-Pyrrolidone). Synthesis, Characterization and Self-Assembly in Aqueous Solution. *J Colloid Interface Sci* **2007**, *310* (1), 136–143. https://doi.org/https://doi.org/10.1016/j.jcis.2007.01.013.

(16) Lee, E. S.; Oh, Y. T.; Youn, Y. S.; Nam, M.; Park, B.; Yun, J.; Kim, J. H.; Song, H.-T.; Oh, K. T. Binary Mixing of Micelles Using Pluronics for a Nano-Sized Drug Delivery System. *Colloids Surf B Biointerfaces* **2011**, *82* (1), 190–195. https://doi.org/https://doi.org/10.1016/j.colsurfb.2010.08.033.

(17) De Lisi, R.; Milioto, S.; Muratore, N. Thermodynamics of Surfactants, Block Copolymers and Their Mixtures in Water: The Role of the Isothermal Calorimetry. *International Journal of Molecular Sciences 2009, Vol. 10, Pages 2873-2895* **2009**, *10* (7), 2873–2895. https://doi.org/10.3390/IJMS10072873.

(18) Kadam, Y.; Singh, K.; Marangoni, D. G.; Ma, J. H.; Aswal, V. K.; Bahadur, P. Thermodynamic of Micelle Formation of Nonlinear Block Co-Polymer Tetronic T904 in Aqueous Salt Solution. *Colloids Surf A Physicochem Eng Asp* **2010**, *369* (1), 121–127. https://doi.org/https://doi.org/10.1016/j.colsurfa.2010.08.010.

(19) Agudelo, Á. J. P.; Coelho, Y. L.; Ferreira, G. M. D.; Ferreira, G. M. D.; Hudson, E. A.; dos Santos Pires, A. C.; da Silva, L. H. M. Solvophobic Effect of 1-Alkyl-3-Methylimidazolium Chloride on the Thermodynamic of Complexation between β-Cyclodextrin and Dodecylpyridinium Cation. *Colloids Surf A Physicochem Eng Asp* **2019**, *582*, 123850. https://doi.org/https://doi.org/10.1016/j.colsurfa.2019.123850.

(20) Loh, W.; Brinatti, C.; Tam, K. C. Use of Isothermal Titration Calorimetry to Study Surfactant Aggregation in Colloidal Systems. *Biochimica et Biophysica Acta (BBA) - General Subjects* **2016**, *1860* (5), 999–1016. https://doi.org/https://doi.org/10.1016/j.bbagen.2015.10.003
